# Grape Exosome–Like Nanovesicles Reverse the Prediabetic State in Mice

**DOI:** 10.1155/jdr/6667696

**Published:** 2026-05-31

**Authors:** Zao-Ling Liu, Shu-Ya Cai, Aerna Qiayimaerdan, Zulipia Zunon, Ya-Le Yu, Xiao-Die Ma

**Affiliations:** ^1^ School of Public Health, Xinjiang Medical University, Urumqi, China, xjmu.edu.cn

**Keywords:** grape exosome–like nanovesicles, gut microbiota, nontargeted metabolomics, prediabetes

## Abstract

**Objective:**

This research explored the effect of grape exosome–like nanovesicles (GELNs) on reversing prediabetic conditions in mice and investigated the underlying mechanisms via metabolomic and gut microbiota analyses.

**Methods:**

Twenty‐four C57BL/6J mice were divided into four groups: normal control, prediabetic model, GELN intervention, and nutrient intervention. Prediabetic models were induced in all but the control group, and then the intervention groups were treated for 8 weeks.

**Results:**

GELNs significantly improved fasting blood glucose, 2‐h postprandial glucose, insulin levels, and total cholesterol. Metabolomic analysis found enriched differential metabolites, highlighting tryptophan and riboflavin metabolism. 16S rDNA sequencing showed no difference in *α*‐diversity but differences in *β*‐diversity. At the phylum level, the abundance of *Firmicutes* and *Desulfobacterota* was higher in the GELN group (Group W), while *Bacteroidota* and *Proteobacteria* were more abundant in the model group (Group T). At the genus level, *Akkermansia* was significantly reduced in the GELN group (Group W), indicating a notable shift in gut microbiota composition.

**Conclusion:**

GELNs may reverse prediabetes in mice by improving metabolic health and gut homeostasis, offering new insights for prediabetes prevention and treatment.

## 1. Introduction

Prediabetes is a metabolic disorder characterized by elevated blood glucose levels that exceed normal thresholds yet remain below the diagnostic criteria for diabetes. This condition represents a critical transitional stage on the pathophysiological continuum toward Type 2 diabetes mellitus (T2DM) [1]. The global prevalence of prediabetes has exhibited a consistent upward trend in recent years, establishing it as a significant public health concern [2]. Empirical evidence indicates that prediabetes constitutes a reversible condition [3], whereby the implementation of timely and targeted interventions can significantly impede or even prevent its progression to T2DM [1]. Therefore, it is imperative to advance our understanding of the underlying pathogenic mechanisms of prediabetes and to devise effective interventional strategies, as these endeavors are critical for the primary prevention and clinical management of T2DM.

While the reduction of blood glucose levels remains a primary therapeutic objective in diabetes management, the adverse effects associated with current hypoglycemic agents have prompted intensive research interest in the development of safe and efficacious plant‐derived alternatives [4]. Accumulating experimental evidence indicates that phytochemicals mediate a wide spectrum of regulatory mechanisms [5]. For instance, polyphenol‐rich plants have been demonstrated to suppress signal transduction pathways mediated by the epidermal growth factor receptor (EGFR) and vascular endothelial growth factor receptor (VEGFR), attenuate inflammatory responses, and modulate epigenetic alterations, thereby exhibiting potential as chemopreventive agents against colorectal cancer [6]. Recent research has revealed that, in addition to the well‐established mechanisms by which phytochemicals influence human physiology, the interspecies regulatory functions of plant microRNAs (miRNAs) constitute a novel pathway that significantly impacts human health through the consumption of plant‐based foods. Owing to their unique methylation patterns, plant miRNAs demonstrate remarkable stability within the gastrointestinal tract and are capable of entering the systemic circulation, where they can target and modulate host gene expression [7]. Building upon existing research, this study provides an in‐depth investigation into the potential application of plant‐derived miRNAs in diabetes intervention, with a specific focus on the feasibility and advantages of utilizing edible plants for prediabetes management. Unlike medicinal plants, edible plants are generally more acceptable for long‐term consumption due to their widespread availability, thereby offering greater translational relevance and practical utility [8]. From a dietary intervention perspective, investigations into edible plant–derived miRNAs are anticipated to yield safer and more effective strategies for the prevention and management of diabetes. This research direction aligns with the health management paradigm positing a shared origin between food and medicine [9]. Numerous studies have established that plant miRNAs can modulate lipid metabolism, suppress viral replication [10], and promote muscle hypertrophy [11], among other physiological functions. For instance, plant miR‐168a has been demonstrated to specifically target and bind to the mRNA of low‐density lipoprotein receptor adapter protein 1 (LDLRAP1), thereby suppressing its expression and mediating interspecies regulation of lipid metabolism in murine models [12]. Furthermore, in addition to the inherent structural stability of plant miRNAs, numerous studies have demonstrated that extracellular carriers, such as exosomes and microvesicles, protect these molecules from nuclease‐mediated degradation and facilitate their delivery into mammalian systems, thereby enabling cross‐kingdom regulatory effects [13]. Researchers have successfully identified and characterized exosome‐like nanovesicles (ELNs) derived from decoctions of plant‐based traditional Chinese medicine. These ELNs are composed of plant‐derived lipids, miRNAs, bioactive compounds, and proteins [14]. Experimental studies conducted both in vitro and in vivo have demonstrated that ELNs derived from *Rhodiola* and *Taraxacum* exhibit significant antifibrotic activity and afford substantial protection against acute lung injury, respectively. Notably, the structural integrity of these ELNs has been shown to protect plant‐derived miRNAs from nuclease‐mediated degradation during cellular internalization and throughout systemic circulation. This protective mechanism enhances the bioavailability of bioactive herbal constituents in the bloodstream, thereby potentiating their therapeutic efficacy [14]. Plant exosome–like nanovesicles (PELNs) exhibit unique biological functions that are dictated by variations in their miRNA repertoire, lipidome profile, and the presence of bioactive constituents. For instance, ELNs isolated from *Zingiber officinale* (ginger) and enriched in osa‐miR164d have been demonstrated to ameliorate colitis‐associated symptoms by orchestrating macrophage polarization [15]. Tea‐derived extracellular vesicles (EVs) have been demonstrated to ameliorate murine inflammatory bowel disease (IBD) by suppressing proinflammatory cytokine production, mitigating oxidative stress, and preserving intestinal microbiota homeostasis [16]. Extracts derived from *Citrus limon* have been demonstrated to induce apoptosis in gastric carcinoma cells via reactive oxygen species (ROS)–mediated mechanisms, indicating their potential utility as a therapeutic agent [17].

Although previous studies have elucidated the potential roles of plant‐derived miRNAs in various disease models, their specific effects and underlying molecular mechanisms in the context of prediabetes intervention remain incompletely understood. This knowledge gap is particularly pronounced in research concerning edible plants, where relevant investigations are notably scarce. Despite limited exploration of plant miRNAs in diabetes research, emerging evidence suggests their potential regulatory functions in insulin resistance and pancreatic *β*‐cell functionality through the modulation of gene expression [18]. Specifically, *Ginkgo biloba* extract (GBE) has been demonstrated to reduce the urinary albumin‐to‐creatinine ratio (UACR) and improve metabolic parameters in patients with diabetic kidney disease (DKD), potentially through the downregulation of exosomal miR‐342‐3p [19]. Therapeutic nanoparticles derived from curcumin have been demonstrated to promote diabetic wound healing through the modulation of multicellular networks, highlighting their potential utility in the field of regenerative medicine [20].

EVs from diverse sources have demonstrated significant potential in promoting diabetic wound healing. In the context of diabetes‐related pathologies, EVs are emerging as a promising therapeutic modality owing to their capacity for tissue repair and regeneration. For instance, bacterial extracellular vesicles (BEVs) derived from *Lactobacillus reuteri* can deliver miR‐21a‐5p, activate the PI3K/AKT signaling pathway, and thereby facilitate diabetic wound repair [21]. Furthermore, lemon‐derived nanoparticle‐functionalized hydrogels can regulate macrophage reprogramming and promote diabetic wound healing [22]. Subsequent studies have demonstrated that ELNs derived from mango and *Z. officinale* (ginger) promote diabetic wound healing by upregulating the expression of the migration‐inducing protein follistatin‐like 1 (FSTL1) [23]. Collectively, these studies indicate that EVs from diverse sources hold significant potential for diabetic wound healing, offering novel insights for the treatment of diabetic complications. However, while research on EVs and nanoparticles derived from medicinal plants is relatively extensive, investigations into commonly consumed plants and their associated miRNAs in diabetes intervention remain sporadic, being limited to specific examples such as lemon, mango, and ginger. Overall, current research in this area is insufficient, and mechanistic studies elucidating their roles are particularly scarce. This paucity of data hinders a comprehensive understanding of the therapeutic potential of plant‐derived miRNAs in diabetes management. Based on these findings, systematic investigation into edible PELNs is urgently warranted. In comparison to their medicinal plant counterparts, grape exosome–like nanovesicles (GELNs) may possess unique transport properties and biological functionalities [24]. Moreover, given the widespread consumption of grapes (*Vitis vinifera* L., vvi), there are currently no reports elucidating whether their miRNAs (vvi‐miRNAs) exert antidiabetic effects and the underlying molecular mechanisms involved. This research gap not only provides a novel entry point for investigating plant‐derived miRNAs in diabetes intervention but also lays the groundwork for developing natural and safe strategies for diabetes management.

The grape, a deciduous vine of the Vitaceae family, produces mature berries that are not only widely consumed as a popular fruit but also hold medicinal value in Uyghur traditional medicine [25]. Specific cultivars, such as “Mamazi” and “Suosuo,” are utilized for their tonic properties, which are purported to enhance physical vitality, resolve hard masses, dispel cold, and promote the optimal function of the liver and gallbladder [25]. Grape fruits and seeds are replete with bioactive compounds, including oleanolic acid, resveratrol, quercetin, and proanthocyanidins. Studies have demonstrated that these phytochemicals exert hypoglycemic and lipid‐modulating effects in both diabetic murine models and human subjects, primarily via their anti‐inflammatory and antioxidant activities [26]. Importantly, these compounds also promote the repair of damaged pancreatic *β*‐cells while regulating their proliferation and apoptosis [26]. To date, few studies have investigated the potential antidiabetic mechanisms of grape‐derived miRNAs (vvi‐miRNAs) from the perspective of cross‐species regulation, representing a notable gap in the literature both domestically and internationally. This study therefore presents a novel investigation into the functional roles of GELNs and their encapsulated miRNAs in prediabetes intervention. Utilizing a robust experimental framework and comprehensive multiomics analyses, we systematically evaluated the metabolic regulatory mechanisms of GELNs in prediabetic murine models. Our findings address this critical research gap regarding grape miRNAs in diabetes intervention and provide novel scientific evidence supporting the potential of edible plants in diabetes prevention.

Nontargeted metabolomics constitutes a systematic analytical approach for the comprehensive characterization of all small‐molecule metabolites within an organism, thereby offering critical insights into the pathophysiological mechanisms underlying metabolic disorders [27]. Metabolomic analysis enables a comprehensive investigation of metabolic network alterations during the prediabetic phase, facilitating the identification of potential therapeutic targets. Furthermore, a healthy gut microbiota maintains a delicate dynamic equilibrium, the disruption of which has been implicated in the pathogenesis of numerous diseases [28–30]. Recent advances in gut microbiota research have revealed a significant association between microbial dysbiosis and the onset of T2DM. Specifically, studies indicate that alterations in gut bacterial composition substantially influence insulin signaling pathways [31]. Consequently, identifying the factors contributing to microbial dysbiosis and restoring intestinal microbiota homeostasis may offer novel strategies for the prevention and management of T2DM. In this study, we comprehensively evaluated the multifaceted effects of GELNs in prediabetic murine models using an integrated approach combining nontargeted metabolomic and gut microbiota analyses. This holistic methodology not only elucidates the underlying mechanisms of GELNs but also provides novel scientific evidence for prediabetes intervention strategies, highlighting the promising potential of plant‐derived exosomes in diabetes management.

This study aims to establish a prediabetic mouse model and implement an antioxidant nutrient intervention group as a positive control. Our preliminary findings suggest that antioxidant nutrients play a pivotal role in alleviating oxidative stress and improving insulin sensitivity, thereby effectively delaying the progression from prediabetes to diabetes. The selection of these antioxidant nutrients is based on prior investigations [32] conducted by our research team. The experimental design comprises several defined groups: a blank control group, a prediabetic model group, an antioxidant nutrient group, and a GELN group. The primary objective of this study is to evaluate whether GELNs can ameliorate the prediabetic state in mice while concurrently investigating the underlying mechanisms via nontargeted metabolomic and gut microbiota analyses. Ultimately, this research aims to provide novel insights for the development of safe and effective botanical therapeutics and to establish a scientific basis for the early prevention and intervention of prediabetes.

## 2. Materials and Methods

### 2.1. Materials

#### 2.1.1. Experimental Animals

A total of 24 male SPF‐grade C57BL/6J mice (14–16 g) were procured from Beijing Vital River Laboratory Animal Technology Co., Ltd. The mice were housed in SPF‐grade facilities at the Experimental Animal Center of Xinjiang Medical University, with production license SCXK (Beijing) 2021‐0006 and use license SCXK (Xinjiang) 2023‐0004. All animal experiments were approved by the Experimental Animal Ethics Committee of Xinjiang Medical University (Approval No. IACUC‐20220720‐25). Housing conditions were strictly controlled: temperature (20°C–22°C), relative humidity (40%–70%), and a 12‐h light/dark cycle. After a 7‐day acclimatization period in the SPF facility, the mice underwent modeling and experimental procedures.

#### 2.1.2. Main Reagents and Equipment

The main reagents used in this study included the following: streptozotocin (STZ) (S0130, Sigma‐Aldrich), GELNs (Wuhan Jinkai Rui Biotechnology Co., Ltd.), triglyceride (TG) assay kit (Nanjing Jiancheng Bioengineering Institute), total cholesterol (TC) assay kit (Nanjing Jiancheng Bioengineering Institute), and mouse insulin enzyme‐linked immunosorbent assay (ELISA) kit (Shanghai Yuyan Biotechnology Co., Ltd.).

The main instruments used in this study included the following: micropipettes (Eppendorf), biosafety cabinet (Thermo Fisher Scientific), benchtop centrifuge (Sigma Laborzentrifugen GmbH), full‐wavelength microplate reader (Thermo Fisher Scientific), ultrahigh‐performance liquid chromatography (UHPLC) system (Thermo Fisher Scientific), and high‐resolution mass spectrometer (Thermo Fisher Scientific).

### 2.2. Methods

#### 2.2.1. Grouping and Animal Model Preparation

##### 2.2.1.1. Animal Grouping and Model Preparation

The mice were randomly assigned to four groups (*n* = 6 per group) as follows:

1. Control group (healthy control): Maintained on a normal diet with no intervention.
2. Model group (prediabetic model control): Induced to a prediabetic state via a high‐sugar, high‐fat (HSHF) diet combined with STZ injection; no additional interventions were administered postmodeling.
3. GELN intervention group: Treated with GELNs via gavage after HSHF diet plus STZ‐induced prediabetic modeling.
4. Nutrient intervention group: Administered a nutrient mixture (vitamin C, vitamin E, and selenium) via gavage following HSHF diet plus STZ‐induced prediabetic modeling. The nutrient selection for this study prioritized compounds with high scores in the antioxidant dietary pattern identified in our research team’s earlier investigations [32].

##### 2.2.1.2. Model Establishment Protocol

The model establishment protocol in this study was adapted from the methodology developed by the Wang Shaoyun team for the “13th Five‐Year” National Key Research and Development Program project [33]. The specific steps are as follows. Mice were maintained in an environment with controlled temperature (20°C–25°C) and relative humidity (45%–65%) and fed a 60% high‐fat diet (HFD) for 3 weeks. Following a 12‐h fast, mice were intraperitoneally injected with STZ at 60 mg/kg. The STZ solution was prepared in a dark ice bath immediately before use and administered within 30 min to ensure stability. On Days 7 and 21 post‐STZ injection, an oral glucose tolerance test (OGTT) was performed: blood was collected from the tail vein, and blood glucose levels were measured. Based on OGTT results, mice with fasting blood glucose (FBG) of < 7.8 mmol/L and 2‐h postprandial blood glucose (2hPG) between 7.8 and 11.1 mmol/L were preliminarily classified as prediabetic. If modeling was unsuccessful, supplementary STZ injections were administered until the prediabetic criteria were met.

##### 2.2.1.3. Postmodeling Treatment Regimens

Following successful model establishment, the treatment protocols for each group were as follows:

1. Control group

Treatment: Maintained on a normal diet with no additional interventions.

Intervention period: No intervention administered.

2. Model group (prediabetic model control)

Treatment: Continued on a 60% HFD without additional interventions.

Intervention period: Weeks 4–12 postmodeling (total duration: 8 weeks).

3. GELN intervention group

Treatment: Continued on a 60% HFD and administered GELNs via daily gavage.

Intervention dose: GELNs (0.5 mg per mouse) suspended in 1‐mL phosphate‐buffered saline (PBS); dosing regimen referenced from Kim et al. [34].

Intervention period: Weeks 4–12 postmodeling (total duration: 8 weeks).

4. Nutrient intervention group

Treatment: Continued on a 60% HFD and administered a nutrient mixture (vitamin C, vitamin E, and selenium) via daily gavage.

Intervention dose: Vitamin C (15.17 mg/kg), vitamin E (2.12 mg/kg), and selenium (9.1 *μ*g/kg). Dosages were converted from adult human reference intakes (per the 2023 edition of the *Dietary Nutrient Reference Intakes for Chinese Residents* compiled by the Chinese Nutrition Society) to murine equivalents using a conversion factor of 9.01 (human‐to‐mouse), with adjustments made daily based on individual mouse body weight.

Intervention period: Weeks 4–12 postmodeling (total duration: 8 weeks).

The specific research design can be found in Table S1 of the Supporting Information section.

#### 2.2.2. Extraction of GELNs

For sample pretreatment, *Maljiazi* grapes (from Xinjiang) were selected. The procedure began with initial weighing, followed by peeling and reweighing. The grapes were then rinsed with ultrapure water and homogenized in PBS, and the resulting juice was filtered through gauze. The filtrate was sequentially centrifuged at 1000 × *g* for 10 min and 3000 × *g* for 30 min, with supernatants collected after each step. The clarified supernatant was further concentrated using a 100‐kDa ultrafiltration device, followed by sequential centrifugations at 3500 × *g* (30 min) and 10,000 × *g* (30 min), with intermediate supernatants collected. The final supernatant was sterile‐filtered through a 0.22‐*μ*m membrane.

For exosome isolation, the pretreated sample was first centrifuged at 100,000 × *g* (4°C, 1 h) to remove cellular debris. The pellet was resuspended in PBS and subjected to a second centrifugation at 100,000 × *g* (4°C, 1 h) to eliminate residual impurities. The washed pellet was then resuspended in an appropriate volume of PBS, and exosomes were recovered from the supernatant via low‐speed centrifugation.

#### 2.2.3. Exosome Sample Observation by Transmission Electron Microscopy (TEM)

Initially, 10 *μ*L of exosomes was deposited onto a carbon‐coated copper grid and allowed to adsorb for 1 min, after which excess liquid was removed using filter paper. The grid was then stained with 10 *μ*L of 2% uranyl acetate (*w*/*v*) for 1 min, followed by blotting to remove unbound stain. After air‐drying at room temperature, TEM images were acquired at 100 kV to characterize exosome morphology.

#### 2.2.4. Particle Size Analysis of Exosome Samples

Cryopreserved samples were rapidly thawed in a 25°C water bath, immediately chilled on ice, and diluted with 1× PBS. The exosome samples were subsequently analyzed by nanoparticle tracking analysis (NTA) to determine their particle size distribution.

#### 2.2.5. Protein Extraction and Concentration Determination of Exosome Samples

Exosomes were gently resuspended at 37°C, followed by the rapid addition of 5× RIPA lysis buffer. The mixture was thoroughly vortexed and incubated on ice for 30 min with intermittent mixing. For protein quantification, standard curves were generated using bicinchoninic acid (BCA) protein assay kit reagents. Sample aliquots (5 *μ*L) were mixed with BCA working solution and incubated at 37°C for 30 min, and absorbance was measured at 562 nm using a microplate reader. Protein concentrations were calculated based on the standard curve.

#### 2.2.6. OGTT

An OGTT was performed on Day 7, Day 21 after model establishment, and 1 week prior to sacrifice. All mice were fasted for 12 h before testing, with free access to water to prevent dehydration. Following intragastric administration of glucose at a dose of 2 g/kg body weight, blood glucose concentrations were measured at 0, 15, 30, 60, and 120 min, and the area under the glucose concentration–time curve (AUC) was calculated.

The procedure was carried out as follows. On the evening preceding the test, the mice were transferred to a clean cage at 21:00 and fasted until 09:00 the following morning, during which they had ad libitum access to drinking water. At 09:00, the OGTT was initiated. Body weight was first recorded, followed by measurement of FBG. Each mouse was gently restrained on a wire mesh, and approximately 1–2 mm of the tail tip was excised. A small drop of blood was obtained by gentle pressure and applied to a blood glucose meter; this value was recorded as the 0‐min blood glucose concentration. Care was taken throughout the procedure to minimize stress to the animals.

After a 30‐min acclimatization period, a glucose solution was prepared for intragastric administration. Using a 1‐mL syringe fitted with a gavage needle, the solution was delivered at a volume of 0.01 mL/g body weight. Timing commenced immediately after administration, and blood glucose was remeasured at 15, 30, 60, 90, and 120 min using the same method described above. Upon completion of the test, food was returned to the cages without delay.

#### 2.2.7. Sample Collection and Processing

The body weight of all mice was systematically recorded at predetermined weekly intervals throughout the study. FBG levels were measured biweekly, and bedding was changed every 3 days to maintain hygiene. In addition, general health assessments were performed regularly, with attention to fur condition, ocular and nasal discharges, the presence of skin lesions or abscesses, locomotor activity, responsiveness to stimuli, and nutritional status. Relevant observations were documented as required.

Following 8 weeks of gavage administration, the mice were subjected to an overnight fast. On the subsequent day, immediately prior to euthanasia, each animal was weighed, and its FBG concentration was determined using a glucometer. Thereafter, blood and tissue samples were collected for biochemical and histological analyses.

#### 2.2.8. Serum Marker Assays

Serum insulin, TC, and TG were quantified in accordance with the manufacturer’s instructions provided with the respective assay kits.

#### 2.2.9. Insulin Resistance Index (HOMA‐IR) and Insulin *β*‐Cell Function Index (HOMA‐*β*)

The insulin resistance index (HOMA‐IR) was calculated using the following equation:

HOMA−IR=FBG×FINS22.5,

where FBG denotes fasting blood glucose (mmol/L) and FINS denotes fasting insulin (*μ*U/mL).

The insulin *β*‐cell function index (HOMA‐*β*) was determined as follows:

HOMA−β=20×FINSFBG−3.5,

where FINS denotes the fasting insulin level, expressed in *μ*IU/mL, and FBG denotes the fasting blood glucose level, expressed in mmol/L. The constant 3.5, also in mmol/L, corresponds to the mean FBG level observed in healthy individuals. The HOMA‐*β* index serves as an indicator of pancreatic *β*‐cell secretory function. Higher values of the index are associated with enhanced insulin secretion capacity, whereas lower values suggest impairment of *β*‐cell function.

#### 2.2.10. Nontargeted Metabolomic Detection and Data Transformation

Untargeted metabolomic analysis of intestinal contents was performed using ultrahigh‐performance liquid chromatography coupled with Orbitrap mass spectrometry (UHPLC‐Orbitrap‐MS).

For each sample, 25 mg of intestinal content was accurately weighed into Eppendorf tubes under controlled low‐temperature conditions (4°C). Homogenization beads and 500 *μ*L of precooled extraction solvent (methanol:acetonitrile:water, 2:2:1, *v*/*v*/*v*) containing isotope‐labeled internal standards were added. Samples were vortexed for 30 s, homogenized at 35 Hz for 4 min, and subjected to three cycles of ultrasonication in an ice‐water bath (5 min per cycle). Following incubation at −40°C for 1 h, samples were centrifuged at 12,000 rpm for 15 min at 4°C to remove particulates. The resulting supernatants were aliquoted for UHPLC‐MS analysis, and pooled quality control samples were prepared by combining equal volumes of each individual extract.

Polar metabolites were separated on a Vanquish UHPLC system (Thermo Fisher Scientific) equipped with an ACQUITY UPLC BEH Amide column (2.1 × 100 mm, 1.7 *μ*m; Waters). The mobile phase consisted of (A) 25 mmol/L ammonium acetate and 25 mmol/L ammonia in water and (B) acetonitrile. The column temperature was maintained at 4°C, and the injection volume was 2 *μ*L.

Nonpolar metabolites were analyzed under the same chromatographic system using a Kinetex C18 column (2.1 × 100 mm, 2.6 *μ*m; Phenomenex) with the mobile phase consisting of (A) 0.01% acetic acid in water and (B) isopropanol:acetonitrile (1:1, *v*/*v*), while keeping the column temperature and injection volume unchanged.

Raw data were converted to mzXML format using ProteoWizard software. Metabolite identification was performed using a custom R package developed in collaboration with the analytical service provider, with reference to the BiotreeDB (v3.0) database. Identification was based on matching experimental mass‐to‐charge ratios (*m*/*z*) and retention times to those of known compounds in the database. Quantification was achieved by constructing calibration curves from reference standards of known concentrations, with peak area or peak height used for concentration calculations. An in‐house R package was employed for downstream statistical analysis and visualization, enabling clear representation of intersample differences in metabolite composition and abundance.

#### 2.2.11. Gut Microbiome Detection

Intestinal contents from the cecum and a segment of the small intestine of the collected mice were analyzed by 16S rDNA next‐generation high‐throughput sequencing, targeting the V3–V4, V4, and V4–V5 hypervariable regions, as well as archaeal sequences. Genomic DNA was extracted from cecal and proximal small intestinal contents using the cetyltrimethylammonium bromide (CTAB) method. The quality and quantity of the extracted DNA were assessed by agarose gel electrophoresis (to evaluate purity and integrity) and UV spectrophotometry (to determine concentration). Purified PCR amplicons were subjected to dual quality control using the Agilent 2100 Bioanalyzer and the Kapa Biosystems Library Quantification Kit (Illumina). Libraries meeting the minimum concentration requirement of 2 nM were selected for indexing. Indexed libraries were normalized, pooled, and denatured with 0.2 N NaOH to generate single‐stranded DNA templates. Sequencing was performed on the Illumina NovaSeq 6000 platform using a 2 × 250 − bp paired‐end configuration and the NovaSeq 6000 SP Reagent Kit (500 cycles) for library preparation and sequencing chemistry.

#### 2.2.12. Detection Methods of miRNAs in GELNs

miRNA identification in ELNs isolated from *Vitis amurensis* was performed by small RNA sequencing (smRNA‐seq) and miRNA sequencing (miRNA‐seq). Initial quality assessment of sequencing libraries was carried out using FastQC, with evaluation of per‐base quality scores, nucleotide composition, and other key metrics to verify data integrity and exclude potential contamination or technical bias. Low‐quality reads and adapter sequences were removed using fastp, which included trimming of terminal N bases, filtering for a minimum Phred quality score of Q20, and removal of adapter contaminants. Cleaned sequences were aligned to the Rfam database using Bowtie to filter out noncoding RNAs, such as ribosomal RNA (rRNA) and transfer RNA (tRNA). The remaining sequences were mapped to the *V. amurensis* reference genome, and small RNA abundance was quantified with reference to miRBase annotations. Putative miRNAs were identified by aligning sequences to mature and precursor miRNA entries in miRBase using the mirDeep2 algorithm, enabling detection of highly expressed vvi‐miRNAs. A literature review was conducted to validate the biological relevance of the identified candidates.

#### 2.2.13. Primer Information and 16S rDNA Project Process

In this study, the V4 hypervariable region of the 16S rRNA gene was selected for PCR amplification due to its high sequence variability and extensive database representation, which facilitate accurate discrimination among microbial taxa. This region has been widely adopted in investigations of microbial community structure.

The primer pair 515F (5 ^′^‐GTGYCAGCMGCCGCGGTAA‐3 ^′^) and 806R (5 ^′^‐GGACTACHVGGGTWTCTAAT‐3 ^′^) was chosen for its broad taxonomic coverage, low amplification bias, and compatibility with multiple sequencing platforms. These primers enable reliable reflection of microbial composition in complex samples. Furthermore, the use of a single primer set simplifies the experimental workflow, reduces cost, and streamlines downstream data analysis. Table 1 shows the primer sequences for each item.

**Table 1:** Primer sequences for each item [13].

Amplified fragment	Primer sequence
V3–V4	341F (5 ^′^‐CCTACGGGNGGCWGCAG‐3 ^′^) 805R (5 ^′^‐GACTACHVGGGTATCTAATCC‐3 ^′^)
V4	515F (5 ^′^‐GTGYCAGCMGCCGCGGTAA‐3 ^′^) 806R (5 ^′^‐GGACTACHVGGGTWTCTAAT‐3 ^′^)
V4–V5	F (5 ^′^‐GTGCCAGCMGCCGCGG‐3 ^′^) R (5 ^′^‐CCGTCAATTCMTTTRAGTTT‐3 ^′^)
Archaea	F (5 ^′^‐GYGCASCAGKCGMGAAW‐3 ^′^) R (5 ^′^‐GGACTACHVGGGTWTCTAAT‐3 ^′^)

Total genomic DNA was extracted from microbial community samples using the CTAB method. DNA integrity was assessed by agarose gel electrophoresis, and concentration was measured with a UV spectrophotometer. Target‐specific primers were designed for PCR amplification, with ultrapure water included as a negative control to minimize the risk of false‐positive amplification. PCR products were verified by 2% agarose gel electrophoresis. Amplicons were purified using AMPure XT magnetic beads and quantified with a Qubit fluorometer. Library quality and concentration were further evaluated using an Agilent 2100 Bioanalyzer in combination with Illumina library quantification kits. Libraries meeting the minimum concentration criterion (≥ 2 nM) were normalized, indexed, and pooled according to their unique index sequences. Indexed libraries were denatured with 0.2 N NaOH to generate single‐stranded DNA templates. Sequencing was performed on an Illumina NovaSeq 6000 platform using a 2 × 250 − bp paired‐end configuration. Raw sequencing data (RawData) were generated after run completion. Paired‐end reads were merged using overlap‐based assembly, followed by quality filtering and chimera removal to produce high‐quality CleanData.

Sequence processing was carried out using the DADA2 (Divisive Amplicon Denoising Algorithm) pipeline. Unlike traditional clustering methods based on sequence similarity, DADA2 employs a dereplication step analogous to 100% identity clustering, generating representative sequences with single‐base resolution. This approach enhances both data accuracy and taxonomic resolution. Following denoising, DADA2 constructs an amplicon sequence variant (ASV) table, functionally equivalent to an operational taxonomic unit (OTU) table, and outputs the corresponding feature sequences. Subsequent analyses included *α*‐ and *β*‐diversity assessments, taxonomic annotation, and differential abundance testing.

#### 2.2.14. Statistical Methods for Microbial Analysis

*α*‐Diversity, reflecting species richness and evenness within individual samples, was assessed using the Chao1 estimator, observed species, Good’s coverage, Shannon index, Simpson index, and Pielou’s evenness.

*β*‐Diversity, evaluating compositional dissimilarities among samples, was examined by principal component analysis (PCA), principal coordinates analysis (PCoA), hierarchical clustering (UPGMA), nonmetric multidimensional scaling (NMDS), analysis of similarities (ANOSIM), and permutational multivariate analysis of variance (PERMANOVA).

### 2.3. Statistical Analysis

All data are expressed as mean ± standard deviation (SD). Intergroup comparisons were performed using two‐way analysis of variance (ANOVA). Where homogeneity of variances was confirmed, pairwise comparisons were conducted using the least significant difference (LSD) test; in cases of heterogeneity, Dunnett’s T3 test was applied. Statistical significance was defined as*p* < 0.05.

Graphical representations, including bar and line charts, were generated using GraphPad Prism 9.0. Statistical analyses were carried out using SPSS 26.0.

## 3. Results

### 3.1. Characterization and Identification of GELNs

**Figure 1:**
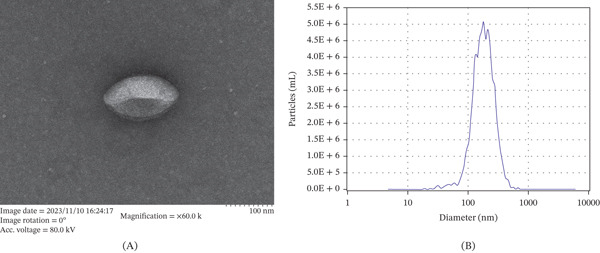
Characterization and identification outcomes of gelatinous GELNs. (A) Transmission electron microscope image of GELNs. (B) Schematic representation of the particle size and concentration of GELNs.

GELNs were examined by TEM at an accelerating voltage of 80 kV (Figure 1A). TEM images revealed irregularly shaped circular or ellipsoidal structures exhibiting central invaginations and distinct dark membranous borders, features characteristic of saucer‐ or cup‐shaped exosomes. NTA was performed to determine particle size distribution and concentration (Figure 1B). The mean vesicle diameter was 193 nm, and the particle concentration was 4.1 × 10^11^ particles/mL. BCA protein assay yielded a vesicular protein concentration of 28.387 *μ*g/*μ*L.

A detailed statistical analysis of the particle size distribution of GELNs was performed, and the results are summarized in Table 2. Table 2 presents particle size values (in nanometers) at the 10th, 50th, and 90th percentiles of the cumulative distribution, together with the corresponding particle numbers, concentrations, and volumes. The standard error of the mean (SEM) for the particle size distribution is also reported, calculated as follows:

SEM=SDn,

where SD is the standard deviation (77.9 nm) and *n* is the number of tracked particles (2528). As shown in Table 2, the particle number, concentration, and volume increased progressively from the 10th percentile to the 90th percentile. Volume exhibited a marked increase with rising particle size. These findings indicate a right‐skewed distribution, with a greater proportion of larger particles. The SD values reflect the dispersion of the size distribution, and the relatively large SD for volume may be attributed to the presence of larger particles.

**Table 2:** Values (all sizes are given in nm).

	Number	Concentration	Volume
X10	106.5	106.5	169.2
X50	177.7	177.7	271.9
X90	288.1	288.1	443.8
Span	1.0	1.0	1.0
Mean	193.0	193.0	297.1
StdDev	77.9	77.9	113.8

### 3.2. Variations in the Types and Expression Levels of miRNAs Within ELNs Derived From Grapes

High‐throughput sequencing of miRNAs from GELNs of *Maljiazi* grapes identified miR11075c‐3p as the most abundant species, with 8804 normalized read counts. This was followed by members of the miR164 family, specifically miR164a‐5p and miR164e‐5p, which showed expression levels of 2604 and 2606 normalized counts, respectively.

**Figure 2:**
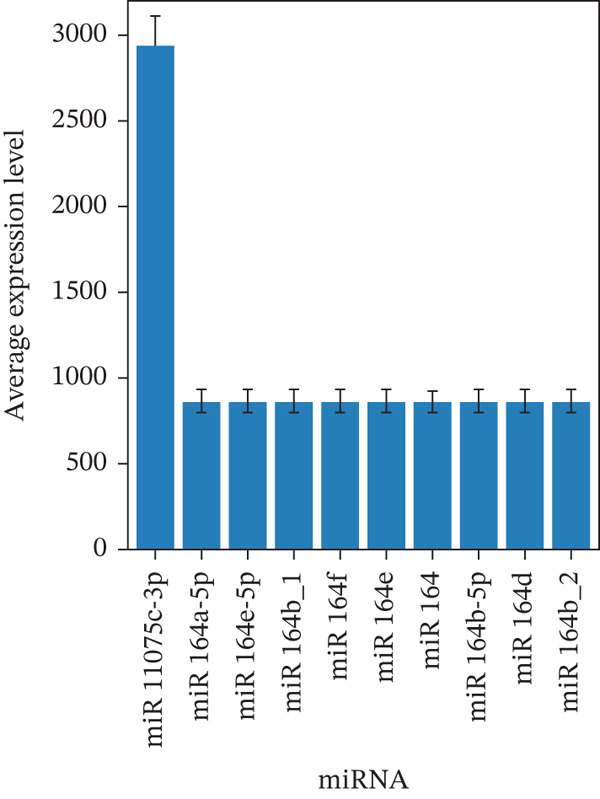
Expression levels of the top 10 miRNAs in the exosome‐like nanovesicles of grapes (*Vitis vinifera* L.).

As shown in Figure 2, the expression profile of the 10 most abundant miRNAs revealed a pronounced predominance of miR11075c‐3p (*p* < 0.001, one‐way ANOVA). While the functional role of miR110 remains to be fully elucidated, recent studies have demonstrated that miR‐164 modulates glucose homeostasis by repressing the insulin signaling pathway through targeting insulin receptor substrate 1 (*IRS1*) [35]. This comprehensive miRNA mapping provides key molecular signatures that are essential for elucidating the biological functions of GELNs.

### 3.3. Impact of GELNs on Fundamental Metrics in a Prediabetic Mouse Model

#### 3.3.1. Effects of Gelatinous GELNs on FBG and 2hPG Levels in a Prediabetic Murine Model

Table 3 summarizes the effects of different treatments on FBG and 2hPG levels in prediabetic mice after an 8‐week gavage period.

**Table 3:** Effects of intervention measures on fasting blood glucose (FBG) and 2‐h postprandial blood glucose (2hPG) levels in murine models exhibiting prediabetic characteristics ($\bar{x}\pm s$).

	Control group (*n* = 6)	Model group (*n* = 6)	GELN group (*n* = 6)	Nutrient group (*n* = 6)	*F*	*p*
FBG	7.75 ± 0.75	13.53 ± 1.43^a^	9.58 ± 0.85^ab^	9.28 ± 0.45^ab^	41.464	< 0.001
2hPG	5.87 ± 0.91	12.27 ± 4.28^a^	9.80 ± 1.12^a^	9.32 ± 1.53^ab^	7.315	0.002

^a^Statistically significant difference when compared to the control group (*p* < 0.05).

^b^Statistically significant difference in relation to the model group (*p* < 0.05).

^c^Statistically significant difference in comparison to the nutrient group (*p* < 0.05).

**Figure 3:**
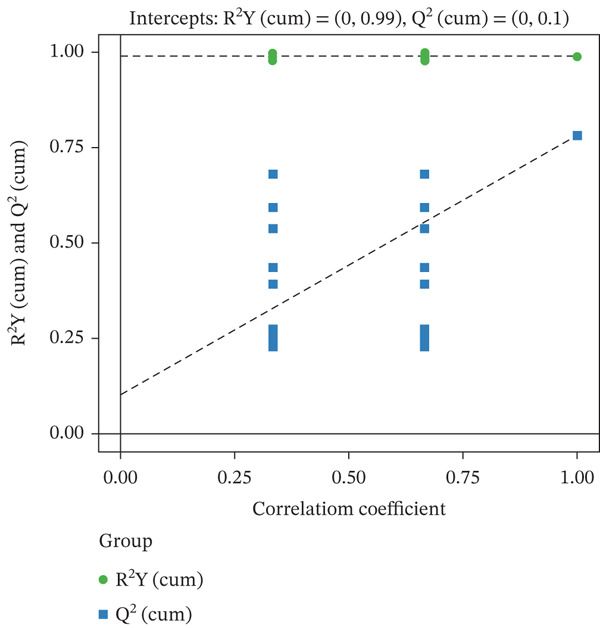
OPLS‐DA permutation plot.

FBG was significantly higher in the model group than in the control group (*p* < 0.05). The GELN group had a mean FBG of 9.58 ± 0.85 mmol/L, which was lower than that of the model group (*p* < 0.05), but remained significantly elevated compared to the control group (*p* < 0.05). Similarly, the nutrient group showed a mean FBG of 9.28 ± 0.45 mmol/L, which was also lower than that of the model group (*p* < 0.05) but higher than that of the control group (*p* < 0.05). For 2hPG, the control group averaged 5.87 ± 0.91 mmol/L, whereas the model group exhibited a significantly higher value of 12.27 ± 4.28 mmol/L (*p* < 0.05). The GELN group had a mean 2hPG of 9.80 ± 1.12 mmol/L, which was lower than that of the model group (*p* < 0.05) but still higher than that of the control group (*p* < 0.05). The nutrient group showed a mean 2hPG of 9.32 ± 1.53 mmol/L, which was also lower than that of the model group (*p* < 0.05) but higher than that of the control group (*p* < 0.05). Figure 3 displays the orthogonal partial least squares discriminant analysis (OPLS‐DA) model, which showed no evidence of overfitting, with a *Q* ^2^ value of 0.781 and an *R* ^2^ *Y* value of 0.989.

#### 3.3.2. Effects of Gelatinous ELNs on FINS, HOMA‐IR, and HOMA‐*β* in a Prediabetic Murine Model

Table 4 summarizes the effects of different treatments on FINS, the insulin resistance index (HOMA‐IR), and the insulin *β*‐cell function index (HOMA‐*β*) in prediabetic mice after an 8‐week gavage period.

**Table 4:** Effects of intervention strategies on FINS, HOMA‐IR, and HOMA‐*β* in mice with prediabetes ($\bar{x}\pm s$).

	Control group (*n* = 6)	Model group (*n* = 6)	GELN group (*n* = 6)	Nutrient group (*n* = 6)	*F*	*p*
FINS (ng/mL)	2.56 ± 0.19	2.25 ± 0.09^a^	2.37 ± 0.17^a^	2.22 ± 0.09^a^	7.147	0.002
HOMA‐IR	23.62 ± 2.78	30.94 ± 17.99	19.76 ± 5.02^b^	19.30 ± 2.68^b^	5.435	0.008
HOMA‐*β*	256.74 ± 33.22	162.06 ± 73.74	332.13 ± 171.43	258.03 ± 70.36	2.851	0.063

^a^Statistically significant difference in comparison to the control group (*p* < 0.05).

^b^Statistically significant difference in comparison to the model group (*p* < 0.05).

^c^Statistically significant difference in comparison to the nutrient group (*p* < 0.05).

The GELN group had a mean FINS level of 2.37 ± 0.17 ng/mL, which was significantly lower than that of the control group (*p* < 0.05). Similarly, the nutrient group showed a mean FINS level of 2.22 ± 0.09 ng/mL, also significantly reduced compared to the control group (*p* < 0.05). Significant differences in HOMA‐IR were observed among groups (*p* < 0.05), whereas no significant differences in HOMA‐*β* were detected (*p* > 0.05).

#### 3.3.3. Influence of Gelatinous ELNs on TC and TG Concentrations in Prediabetic Murine Models

Table 5 presents the effects of different treatments on serum TG and TC levels in prediabetic mice.

**Table 5:** Effects of interventions on total cholesterol and triglyceride levels in prediabetic murine models ($\bar{x}\pm s$).

	Control group (*n* = 6)	Model group (*n* = 6)	GELN group (*n* = 6)	Nutrient group (*n* = 6)	*F*	*p*
TG (mmol/L)	1.02 ± 0.24	0.84 ± 0.23	0.75 ± 0.10	0.71 ± 0.07	2.654	0.082
TC (mmol/L)	2.71 ± 0.13	3.08 ± 0.36	3.57 ± 0.25^abc^	2.63 ± 0.47^b^	8.027	0.002

^a^Statistically significant difference in comparison to the control group (*p* < 0.05).

^b^Statistically significant difference in comparison to the model group (*p* < 0.05).

^c^Statistically significant difference in comparison to the nutrient group (*p* < 0.05).

No statistically significant differences in TG levels were observed among the groups (*p* > 0.05). In contrast, TC levels differed significantly across groups (*F* = 8.027, *p* = 0.002). Specifically, both the model group and the GELN group exhibited higher TC levels than the control group (*p* < 0.05). Additionally, the nutrient group showed lower TC levels compared with the model group (*p* < 0.05).

### 3.4. Impact of Gelatinous ELNs on the Metabolomic Profile in Mouse Models of Prediabetes

#### 3.4.1. Assessment of Differential Metabolites

Following metabolite identification, multivariate statistical analysis was performed on the acquired data. Metabolites were selected based on two criteria: (1) a *p* value from Student’s *t*‐test of less than 0.05 and (2) a variable importance in projection (VIP) value from the first principal component of the OPLS‐DA model greater than 1. The results of metabolite screening for the GELN intervention group (Group W) and the model group (Group T) are listed in Table 6 (first 10 rows). The corresponding OPLS‐DA score plot is shown in the figure. No evidence of overfitting was observed, with *Q* ^2^ = 0.781 and *R* ^2^ *Y* = 0.989.

**Table 6:** Results of differential metabolite screening between Group T and Group W (top 10 entries).

MS2 name	MS2 score	VIP	*p*value	Fold change	Log fold change
Pentadecanoic acid	3.990	1.029	0.022	0.311	−1.684
Valeric acid	3.990	1.829	0.038	0.326	−1.619
Isovaleric acid	3.980	1.829	0.038	0.326	−1.619
Riboflavin	3.970	1.029	0.037	2.560	1.356
LPE (14:0)	3.950	1.398	0.017	0.280	−1.836
Azelaic acid	3.940	1.028	0.014	0.293	−1.771
4‐Hydroxyphenylacetic acid	3.940	1.348	0.012	0.306	−1.706
Kynurenic acid	3.930	1.245	0.003	0.237	−2.078
Phenylacetylglycine	3.920	1.337	0.039	0.208	−2.264
4‐(Dimethylamino)butanoate	3.890	1.215	< 0.001	0.241	−2.056

#### 3.4.2. Differential Metabolite Volcano Plot Analysis

**Figure 4:**
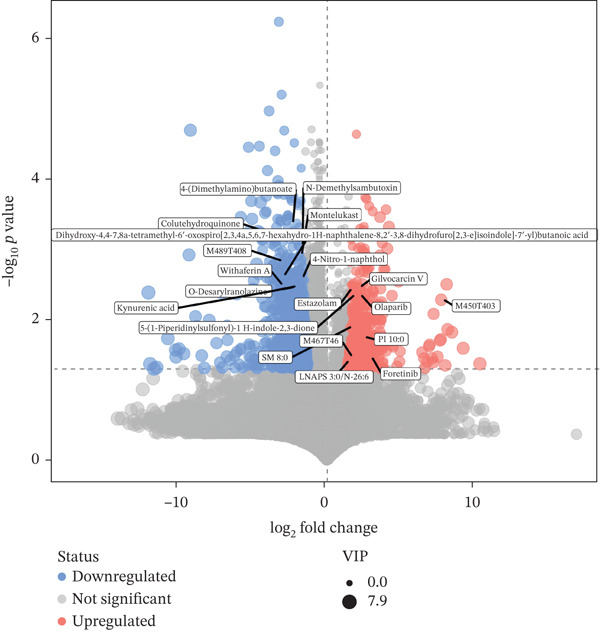
Volcano plot of differential metabolites between Group T and Group W.

The volcano plot serves as an effective visual representation of the overall distribution of metabolic variations between different groups. In comparison to the model group, the GELN intervention group exhibited a significant increase in 1647 metabolites (*p* < 0.05) and a notable decrease in 425 metabolites (*p* < 0.05), as illustrated in Figure 4. The 10 metabolites demonstrating the most significant differential expression are as follows: 5‐[(aminooxy)sulfonyl]‐2,4‐dichlorobenzoic acid (downregulated), 4‐deoxy‐*β*‐D‐gluc‐4‐enuronosyl‐(1,3)‐N‐acetyl‐D‐galactosamine (downregulated), 2‐methylbutanoyl‐CoA (downregulated), CDP‐choline (cytidine diphosphate choline) (downregulated), flufecetin (downregulated), 2‐[(3S,5R)‐5‐[6‐(2,4‐dichlorophenyl)hexyl]‐3‐hydroxy‐2‐oxooxolan‐3‐yl]acetic acid (downregulated), pelargonidin 3‐O‐(6‐O‐malonyl‐*β*‐D‐glucoside) (downregulated), 28‐norbrassinolide (upregulated), S‐(1,2‐dichlorovinyl)‐glutathione (downregulated), and 1‐alkyl‐2‐acylglycerophosphoethanolamine (downregulated).

#### 3.4.3. Kyoto Encyclopedia of Genes and Genomes (KEGG) Enrichment Analysis of Differential Metabolites

Differential metabolites were mapped onto the KEGG pathway database. Based on KEGG pathway annotation, enrichment analysis results were categorized for each group.

**Figure 5:**
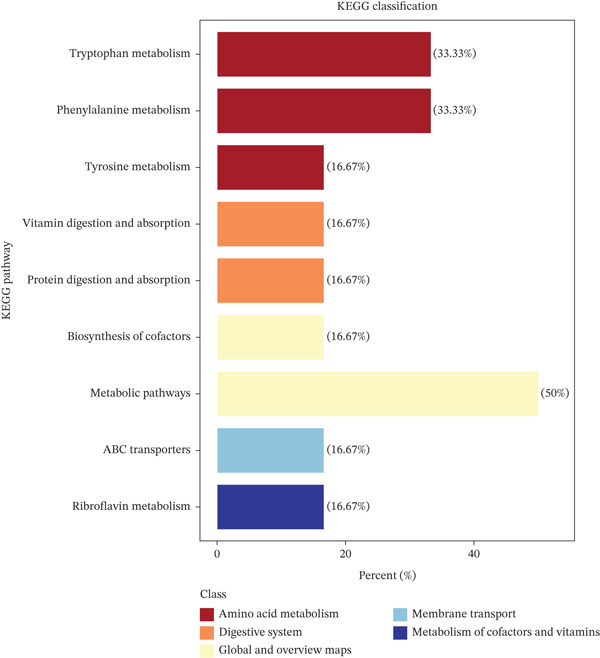
KEGG pathway diagram.

As shown in Figure 5, compared with the model group (Group T), the GELN intervention group (Group W) exhibited significant enrichment of differential metabolites in three pathways: metabolic pathways, phenylalanine metabolism, and tryptophan metabolism. Pathway mapping diagrams for these three metabolic pathways are presented in Figure S1A–C.

#### 3.4.4. Regulatory Network Analysis of Differential Metabolites

After obtaining matched data for differential metabolites from each comparative group, pathway analysis and regulatory interaction network assessment were performed using the KEGG database for *Mus musculus* (mouse). Detailed results are summarized in Table 7.

**Table 7:** Analysis of regulatory networks of differential metabolites.

KEGG ID	Entry type	KEGG name	*p*score
MMU00740	Pathway	Riboflavin metabolism	0.000005
MMU00360	Pathway	Phenylalanine metabolism	0.000148
MMU00380	Pathway	Tryptophan metabolism	0.000976
MMU04977	Pathway	Vitamin digestion and absorption	0.003736

**Figure 6:**
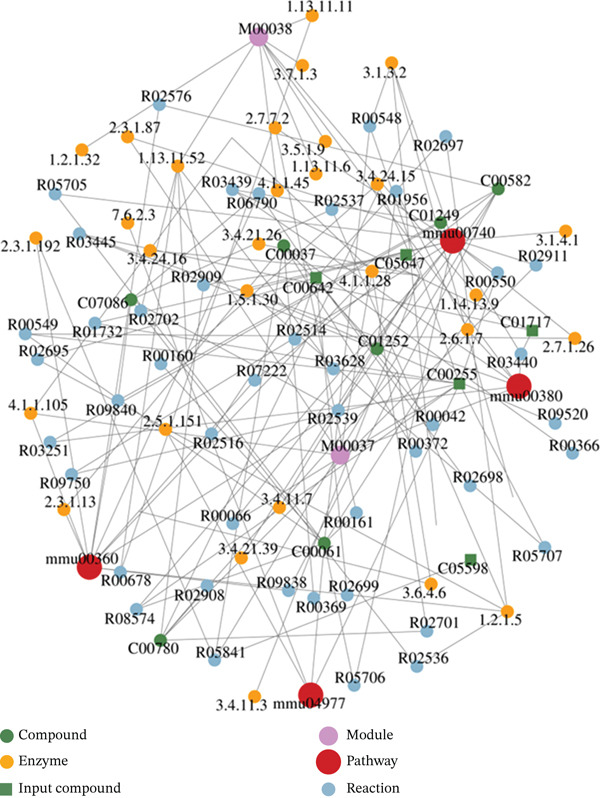
Regulatory network analysis diagram of Group T versus Group W.

As shown in Figure 6, the riboflavin metabolism pathway (MMU00740) occupied a central position in the regulatory network differentiating the model group (Group T) from the GELN intervention group (Group W), displaying multiple connections to other network nodes.

### 3.5. Impact of GELNs on the Gut Microbiota of Prediabetic Mouse Models

#### 3.5.1. Analysis of Gut Microbiota Diversity

*α*‐Diversity analysis was performed to assess species richness and diversity within individual samples. The Chao1 index, Shannon index, and Simpson index were used to evaluate species richness, diversity, and evenness, respectively.

No statistically significant differences were observed among groups for the *α*‐diversity indices—Chao1, observed species, Shannon, and Simpson (*p* > 0.05)—indicating comparable microbial richness and diversity across groups. Detailed results are presented in Table 8.

**Table 8:** *α*‐Diversity index.

*α*‐Diversity index	Group S (*n* = 3)	Group T (*n* = 3)	Group W (*n* = 3)	*F*	*p*
Observed	783.33 ± 199.83	924.00 ± 180.47	874.67 ± 140.91	1.396	0.313
Shannon	6.85 ± 1.07	6.92 ± 1.12	7.44 ± 0.09	0.329	0.805
Simpson	0.95 ± 0.04	0.94 ± 0.08	0.98 ± 0.01	0.471	0.711
Chao1	783.92 ± 199.42	925.73 ± 182.31	875.23 ± 141.36	1.391	0.314

*β*‐Diversity reflects differences in species composition among distinct microbial communities and, together with *α*‐diversity, contributes to the overall biological heterogeneity of a given environment. To reduce the complexity of microbial data and visualize major patterns of variation, methods—specifically PCoA and NMDS—were employed, with samples represented along continuous ordination axes.

**Figure 7:**
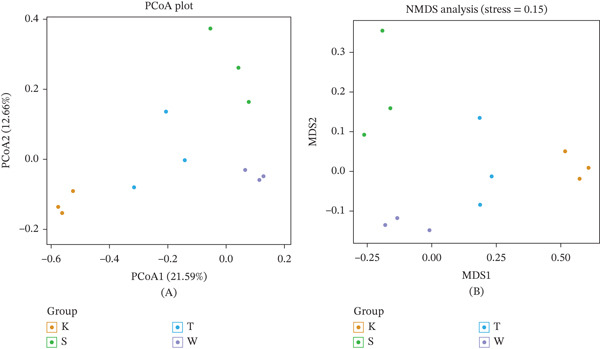
PCoA and NMDS plots. (A) Principal coordinates analysis. (B) Nonmetric multidimensional scaling analysis.

PCoA was performed using Bray–Curtis distance, and the results are shown in Figure 7. The model group (Group T) was markedly separated from the other groups, indicating distinct gut microbiota composition in mice from the model group compared with that in the control and treated groups. NMDS analysis was conducted to confirm these findings; as illustrated in the figure, the NMDS results were consistent with those of the PCoA, supporting the presence of significant differences in gut microbiota composition between the model group and the other groups.

#### 3.5.2. Microbial Community Composition Analysis

**Figure 8:**
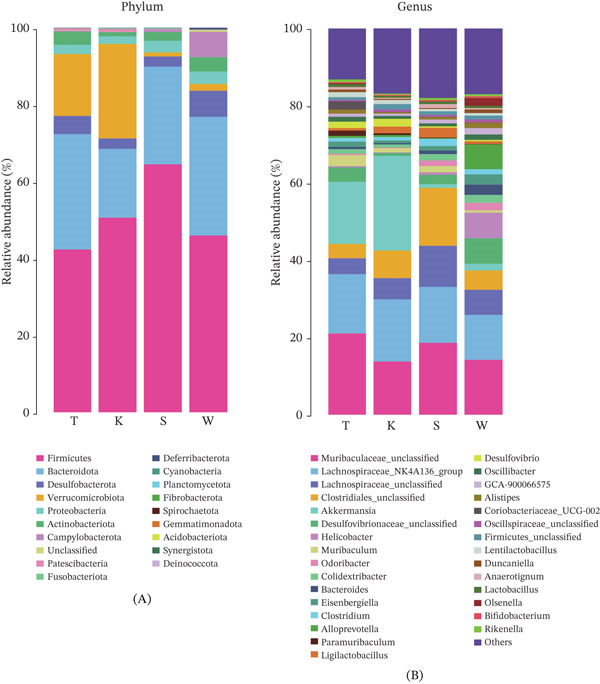
Stacked bar plots of the (A) phylum and (B) genus levels by group.

Figure 8 illustrates the relative abundance of microbial communities at the phylum and genus levels across groups, based on species abundance and annotation data.

At the phylum level, bacterial composition was broadly similar among groups, with the 10 most abundant phyla being *Firmicutes*, *Bacteroidota*, *Desulfobacterota*, *Verrucomicrobiota*, *Proteobacteria*, *Actinobacteriota*, *Campylobacterota*, unclassified bacteria, *Patescibacteria*, and *Fusobacteriota*. Comparison of the five most abundant phyla revealed that *Firmicutes* and *Desulfobacterota* were moderately more abundant in the GELN intervention group (Group W) than in the model group (Group T), whereas *Bacteroidota* and *Proteobacteria* were more prevalent in Group T. Notably, *Verrucomicrobiota* was significantly more abundant in Group T than in Group W, suggesting that GELN intervention reduced the relative abundance of this phylum.

At the genus level, the 10 most abundant taxa included *Muribaculaceae_unclassified*, *Lachnospiraceae_NK4A136_group*, *Lachnospiraceae_unclassified*, *Clostridiales_unclassified*, *Akkermansia*, *Desulfovibrionaceae_unclassified*, *Helicobacter*, *Muribaculum*, *Odoribacter*, and *Colidextribacter*. Among the five most abundant genera, unclassified *Lachnospiraceae* and unclassified *Clostridiales* were slightly more abundant in Group W, whereas unclassified *Muribaculaceae*, *Lachnospiraceae*_*NK4A136_group*, and *Akkermansia* were less abundant in Group W compared with Group T.

#### 3.5.3. Analysis of Intergroup Microbial Community Differences

**Figure 9:**
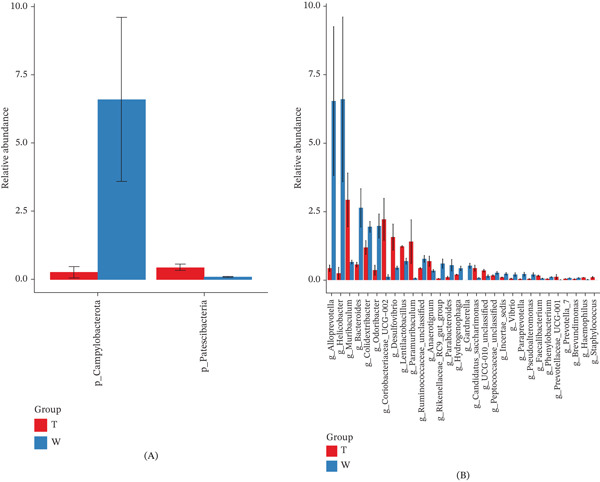
Differences in bacterial communities at the (A) phylum and (B) genus levels between Group W and Group T.

As shown in Figure 9, at the phylum level, *Campylobacterota* (Campylobacteria) was significantly upregulated (*p* < 0.05) and *Patescibacteria* was significantly downregulated (*p* < 0.05) in the GELN intervention group (Group W) compared with the model group (Group T). At the genus level, Group W exhibited a significant increase in the abundance of *Alloprevotella*, *Helicobacter*, *Bacteroides*, *Colidextribacter*, *Odoribacter*, *Ruminococcaceae_unclassified*, *Rikenellaceae_RC9_gut_group*, *Parabacteroides*, *Hydrogenophaga*, *Gardnerella*, *Peptococcaceae_unclassified*, *Incertae_Sedis*, *Vibrio*, *Paraprevotella*, *Pseudoalteromonas*, and *Phenylobacterium* (*p* < 0.05) relative to Group T. Conversely, the abundance of *Muribaculum*, *Coriobacteriaceae*_*UCG-002*, *Desulfovibrio*, *Lentilactobacillus*, *Paramuribaculum*, *Anaerotignum*, *Candidatus_Saccharimonas*, *UCG-010_unclassified*, *Faecalibacterium*, *Prevotellaceae_UCG-001*, *Haemophilus*, and *Staphylococcus* was significantly decreased (*p* < 0.05) in Group W compared with Group T.

#### 3.5.4. Microbial Metabolic Function Prediction

PICRUSt (Phylogenetic Investigation of Communities by Reconstruction of Unobserved States) enables inference of microbial community functions from taxonomic profiles, making it a widely used tool for functional prediction. Using functional prediction results from PICRUSt2, gene family annotations were obtained from public databases, including the Clusters of Orthologous Groups (COG) and KEGG Orthology (KO) databases.

Analysis of COG‐based annotations revealed significant differences in several predicted functional categories between the GELN intervention group (Group W) and the model group (Group T), including exonuclease VII small subunit, leucyl aminopeptidase (aminopeptidase T), and the predicted nucleotide‐utilizing enzyme MoeA. These findings are illustrated in Figure S2.

## 4. Discussion

In this study, miR11075c‐3p was identified as one of the most abundantly expressed miRNAs in ELNs isolated from horse milk seeds, suggesting a potentially important role in their biological functions. Although specific functional roles for miR11075c‐3p have not yet been reported, its high expression level implies possible regulation of cellular processes through targeting of specific genes. Future studies will aim to identify miR11075c‐3p target genes and elucidate its mechanistic involvement in horse milk seed ELNs, thereby providing insight into its biological significance.

### 4.1. Effects of GELNs on Blood Glucose and Related Indicators in Mice

In the model group, FBG and 2hPG levels were significantly elevated compared with the control group. Both the GELN and nutrient intervention groups exhibited lower FBG and 2hPG levels than the model group, although values remained higher than those in the control group. These results indicate that GELN and nutrient interventions partially ameliorated hyperglycemia in prediabetic mice but did not restore glucose levels to normal ranges. FINS levels were significantly reduced in the GELN group relative to the model group, suggesting an effective lowering of serum insulin concentration. In agreement with these observations, Mu et al. [36] reported similar improvements in glycemic control and insulin sensitivity following comparable interventions, demonstrating that PELNs enhance insulin sensitivity through miR‐375‐mediated suppression of the aryl hydrocarbon receptor (AhR). Based on these findings, we hypothesize that GELNs may similarly improve insulin sensitivity via comparable mechanisms.

In the GELN group, a significant reduction in HOMA‐IR was observed compared with the model group, further supporting the potential of GELNs to alleviate insulin resistance. Consistent with this finding, He et al. [37] reported that mung bean exosome–like nanovesicles (MELNs) can mitigate insulin resistance in murine models of T2DM via activation of the PI3K/AKT signaling pathway. Although MELNs and GELNs may share mechanistic similarities, the specific molecular pathways involved in GELN‐mediated effects remain to be elucidated and warrant further investigation through phosphoproteomic profiling and pathway enrichment analysis.

In addition, GELN intervention resulted in elevated TC levels, suggesting a possible exacerbation of lipid metabolism dysregulation in prediabetic mice. This observation aligns with the findings of Wang et al. [38], who demonstrated that exosomes can disrupt lipid metabolism by modulating lipid synthesis, transport, and degradation pathways. In parallel, Wang et al. [39] highlighted the critical role of reverse cholesterol transport (RCT)—primarily mediated by ATP‐binding cassette transporters ABCA1 and ABCG1—in maintaining cholesterol homeostasis. It has been proposed that circulating miRNAs within exosomes, such as miR‐30e and miR‐92a, may suppress ABCA1 and ABCG1 activity, leading to cholesterol accumulation and consequent elevation of TC levels. These findings highlight the imperative for further exploration of the effects of GELNs on lipid metabolism.

### 4.2. Effects of GELNs on Mouse Metabolomics

Compared with the control group, most of the top 10 differential metabolites identified in the grape exosome intervention group possess anti‐inflammatory, antioxidant, and metabolic regulatory properties. CDP‐choline, a key intermediate in phospholipid biosynthesis for cell membranes, was downregulated in the intervention group. This downregulation may indirectly affect the insulin signaling pathway by altering membrane integrity and fluidity, thereby improving insulin sensitivity. Previous studies have shown that CDP‐choline can ameliorate sodium arsenite–induced glucose intolerance, enhance hepatic antioxidant enzyme activity, reduce oxidative stress, and lower levels of proinflammatory cytokines such as tumor necrosis factor‐alpha (TNF‐*α*) and interleukin‐6 (IL‐6) [40]. The results suggest that the anti‐inflammatory properties of CDP‐choline may enhance insulin sensitivity indirectly by alleviating chronic inflammation, which could in turn lead to reduced blood glucose levels. Pelargonidin 3‐O‐(6‐O‐malonyl‐*β*‐D‐glucoside), an anthocyanin derivative, is known for its antioxidant and anti‐inflammatory activities, which may also contribute to improved glycemic regulation [41]. In addition, S‐(1,2‐dichlorovinyl)‐glutathione and its derivatives may modulate blood glucose levels indirectly by mitigating oxidative stress and inflammation [42]. These metabolic alterations point to potential underlying mechanisms; however, further experimental validation is required.

KEGG enrichment analysis revealed that the significant differential metabolites were primarily associated with the metabolic pathways and tryptophan metabolism categories. The broad representation of diverse metabolic pathways suggests that grape exosomes may exert their physiological effects through multiple metabolic regulatory mechanisms. Tryptophan is not only an essential amino acid but also a precursor for several key bioactive compounds, including 5‐hydroxytryptamine (serotonin), indole‐derived metabolites, and nicotinamide. Studies have demonstrated that tryptophan metabolism is closely linked to the pathogenesis and progression of diabetes [43]. For example, the tryptophan metabolite indole‐3‐acetic acid (IPA) possesses anti‐inflammatory properties and can ameliorate insulin resistance, whereas nicotinamide plays a role in insulin secretion and glucose homeostasis [44]. These findings suggest that GELNs may improve insulin resistance by modulating tryptophan metabolism. Our results indicate that GELNs can regulate tryptophan metabolic pathways, potentially through effects on these pivotal metabolites.

Regulatory network analysis of differential metabolites revealed that the riboflavin metabolism pathway (MMU00740) occupies a central position, with extensive connectivity to other network nodes. Riboflavin (vitamin B_2_) plays a vital role in energy and glycolipid metabolism via its active derivatives flavin adenine dinucleotide (FAD) and flavin mononucleotide (FMN). Deficiency in riboflavin may impair mitochondrial function and disrupt tryptophan metabolism, potentially contributing to diabetes development [45]. Accordingly, gelatinous GELNs may ameliorate diabetes‐associated metabolic disorders by modulating riboflavin metabolism or by directly supplementing riboflavin.

### 4.3. Effects of GELNs on the Intestinal Microbiota of Mice

This study evaluated the effects of different treatments on microbial community structure using *α*‐ and *β*‐diversity analyses. Results demonstrated stable *α*‐diversity across groups, alongside distinct *β*‐diversity differentiation. *α*‐Diversity analysis revealed no significant differences in observed species richness, Shannon index (species diversity), or Simpson index (species evenness) among groups, indicating that treatments did not alter overall community richness or species distribution uniformity. This stability may reflect either insufficient treatment intensity/duration to surpass ecological thresholds or tolerance of most species to interventions, such that only minor shifts in rare taxa occurred—insufficient to impact aggregate diversity metrics. Communities likely maintained internal stability via intrinsic regulatory mechanisms (e.g., compensatory growth of tolerant species).

In contrast, PCoA and NMDS of *β*‐diversity revealed significant differences in community composition among groups, with the model group exhibiting marked divergence from other groups. This pattern suggests treatments exerted selective, rather than global, effects: instead of causing widespread species loss or gain, treatments likely altered the relative abundance of dominant taxa, drove shifts in specific functional groups, or triggered ecological niche replacement by modifying resource availability in the microenvironment. Thus, community composition was reshaped without disrupting overall diversity indices. Collectively, this study confirms that while treatments did not affect microbial community richness or evenness, they significantly altered community composition. These findings provide a foundation for understanding microbial responses to environmental perturbations. Further research is warranted to elucidate the molecular mechanisms underlying this compositional differentiation and to assess its potential impacts on host health and ecosystem function.

Analysis of intestinal microbial community composition revealed subtle but distinct phylum‐level shifts associated with treatments. In the GELN intervention group (Group W), the relative abundances of *Firmicutes* and *Desulfobacterota* were moderately elevated compared with the model group (Group T), whereas *Bacteroidota* and *Proteobacteria* were more abundant in Group T. These findings suggest that interventions may selectively modulate the abundance of specific microbial phyla, potentially influencing host metabolism and health status through phylum‐specific functional contributions.

The phylum *Firmicutes* constitutes a dominant component of the intestinal microbiota and exerts a pivotal influence on host energy metabolism and gastrointestinal homeostasis through the production of short‐chain fatty acids (SCFAs). Consequently, supplementation with GELNs may positively modulate host energy metabolism and intestinal health by altering the relative abundance of *Firmicutes*. In the present study, the *Firmicutes*‐to‐*Bacteroidetes* (F/B) ratio was significantly elevated in the GELN‐treated group compared with the model group; however, the biological implications of this observation warrant cautious interpretation.

*Firmicutes* and *Bacteroidota* represent the two most abundant phyla in the gut microbial community, yet distinct genera within the same phylum can exhibit markedly divergent functional roles. Relying solely on the F/B ratio without considering interspecific functional heterogeneity may yield inconsistent or even contradictory findings across studies. For instance, *Faecalibacterium prausnitzii*, a prominent butyrate‐producing species within the *Firmicutes* phylum, plays a critical role in maintaining intestinal health. Butyrate produced by this organism activates the GPR109A receptor on colonocytes, thereby suppressing the NF‐*κ*B signaling pathway and reducing the secretion of proinflammatory cytokines such as IL‐6 and TNF‐*α* [46]. Additionally, butyrate enhances insulin sensitivity by activating G protein–coupled receptors GPR43 and GPR41 and by inhibiting histone deacetylase (HDAC) activity [47]. *Staphylococcus aureus*, a Gram‐positive bacterium belonging to the phylum *Firmicutes*, possesses the capacity to produce enterotoxins that disrupt intestinal epithelial integrity, provoke systemic inflammatory responses, and activate Toll‐like receptor 2 (TLR2). TLR2 engagement promotes macrophage infiltration into adipose tissue, thereby exacerbating insulin resistance [48]. Within the phylum *Bacteroidota*, functional heterogeneity among taxa is evident: *Bacteroides vulgatus* has been reported to suppress glucagon‐like peptide‐1 (GLP‐1) secretion and impair pancreatic *β*‐cell insulin secretory capacity [49]. In contrast, *Bacteroides dorei* contributes to bile acid homeostasis by activating the farnesoid X receptor (FXR), which in turn improves systemic insulin sensitivity [50]. Given these divergent microbial effects on host metabolism, future investigations should incorporate quantitative profiling of SCFAs—particularly butyrate and propionate—to delineate the functional consequences of compositional shifts observed in the intestinal microbiota.

The relative abundance of *Alloprevotella* was markedly elevated in the GELN‐treated group. As a core member of the healthy human gut microbiota, *Alloprevotella* plays a critical role in preserving intestinal homeostasis. Under pathological conditions, its abundance typically declines; thus, the observed increase following GELN administration suggests a potential beneficial effect on prediabetic status. However, the specific mechanisms through which *Alloprevotella* confers metabolic benefits remain to be fully elucidated [51]. In contrast, the genus *Helicobacter* exhibited a significant rise in abundance in the GELN group, indicative of a possible deterioration in disease phenotype. This finding appears to contradict earlier reports in the literature. Previous studies have demonstrated that *Helicobacter pylori* infection promotes hepatic insulin resistance via activation of the c‐Jun/miR‐203/SOCS3 signaling axis [52]. Furthermore, work by Talebi et al. [53] corroborated this association, showing that EVs secreted by two *Helicobacter* strains downregulate key insulin signaling components—including IRS1, AKT2, and GLUT2—while concurrently upregulating proinflammatory mediators (IL‐6, SOCS3, and c‐Jun) and miR‐140, thereby impairing glucose homeostasis. The discrepancy between these results and the current data may be attributable to the relatively short experimental duration (8 weeks) and the limited sample size, underscoring the necessity for larger, longer‐term studies to validate these observations.

At the genus level, the unclassified members of the family *Lachnospiraceae* (*Lachnospiraceae_unclassified*) and the order *Clostridiales* (*Clostridiales_unclassified*) exhibited modestly higher relative abundances in the GELN‐treated group (Group W), potentially indicating a beneficial influence of GELN administration on these microbial communities. In contrast, the unclassified *Muribaculaceae* (*Muribaculaceae_unclassified*), the *Lachnospiraceae* group NK4A136 (*Lachnospiraceae_NK4A136_group*), and the genus *Akkermansia* were present at slightly lower levels in Group W, suggesting that GELN treatment may exert differential effects on these taxa. Notably, the observed decrease in *Akkermansia* abundance in the GELN group appears to conflict with the prevailing view that this genus is positively associated with enhanced glucose tolerance [54]. *Akkermansia muciniphila*, a mucin‐degrading bacterium, has been the focus of extensive research and is well established to promote metabolic health by stimulating mucin turnover in the intestine, which in turn enhances insulin sensitivity [55]. However, the present findings revealed a reduction in *Akkermansia* abundance following GELN intervention, suggesting either a potential suppressive effect of GELNs on this taxon or the existence of complex interactions between *Akkermansia* and the bioactive components of GELNs. Previous studies have demonstrated that PELNs can be selectively internalized by specific gut bacteria, leading to alterations in their metabolic profiles and outer membrane vesicle composition. These changes may subsequently enhance mucosal barrier function, mitigate inflammation, and improve host metabolic parameters via bioactive mediators such as indole derivatives and outer membrane proteins [56]. Furthermore, existing evidence indicates that *Akkermansia* frequently establishes functional consortia with specific co‐occurring bacterial taxa, collaboratively modulating inflammatory responses and metabolic processes [57]. The observed reduction in *Akkermansia* abundance following GELN intervention may reflect competitive resource utilization or ecological niche displacement involving other beneficial taxa. Nevertheless, the overall effect of GELNs appears to promote intestinal barrier integrity and attenuate inflammation through remodeling of the microbial community structure and metabolite profile [58]. Future studies employing microbiota transplantation, monocolonization experiments, or coculture systems could provide deeper insights into the interactions between GELNs and *Akkermansia*, as well as other functionally relevant gut microbes. In conclusion, GELN intervention exerted discernible effects on specific taxa within the intestinal microbiota, and these alterations may be mechanistically linked to host metabolic status and overall health. Further investigations are warranted to elucidate the underlying mechanisms driving these microbial shifts and to assess their long‐term physiological consequences. Moreover, given the constraints imposed by the relatively small sample size, which may limit the generalizability of the observed microbial changes, future studies should employ larger cohorts to strengthen the robustness, reliability, and reproducibility of the findings.

Analysis of intergroup microbial variation revealed a marked decrease in *Faecalibacterium* abundance in the GELN‐treated group, a finding that contrasts with the well‐documented protective role of this genus in T2DM [59]. *F. prausnitzii*, the most extensively studied species within this genus, is known to fortify intestinal barrier function and regulate immune responses through the production of SCFAs, notably butyrate. However, the observed reduction in *Faecalibacterium* abundance in the present study may be attributable to several factors. First, exosome intervention could have modified the intestinal microenvironment—such as pH, nutrient availability, or redox balance—thereby impeding the proliferation of this genus. Second, the 8‐week experimental duration may have been insufficient to detect substantial shifts in *Faecalibacterium* levels. Finally, the limited sample size constrains the generalizability of these findings, necessitating validation in larger cohorts to ensure the robustness and reliability of the conclusions. Furthermore, the reduced abundance of *Lentilactobacillus* in the GELN‐treated group suggests a limited capacity to alleviate prediabetic phenotypes. Previous studies have reported that supplementation with *Lactiplantibacillus* species significantly lowers FBG and glycated hemoglobin (HbA1c) levels [60]; however, such interventions do not appear to exert a pronounced effect on insulin resistance or lipid metabolism.

Predictive analysis of microbial metabolic function revealed that, relative to the model group, the GELN‐treated group displayed elevated activities of leucyl aminopeptidase and nucleotide‐utilizing enzymes, reflecting an upregulation of proteolytic and nucleotide metabolic pathways. These results are consistent with metabolomic data, which demonstrated significant enrichment of multiple metabolic pathways in the GELN group, indicative of enhanced microbial engagement in fundamental metabolic processes and a potential increase in the synthesis or catabolism of key metabolites. Although PICRUSt2 predictions did not directly identify enzymes involved in tryptophan metabolism, the observed intensification of nucleotide‐related metabolic activity may suggest indirect modulation of tryptophan metabolism. This hypothesis is supported by the central roles of tryptophan‐derived metabolites—such as nicotinic acid and NAD^+^, both of which are integral to nucleotide metabolism—in the regulation of host metabolism [61]. Collectively, these findings suggest that GELN‐mediated alterations in tryptophan‐associated metabolic pathways may interact synergistically with changes in nucleotide metabolism, thereby influencing overall microbial metabolic activity.

In conclusion, GELNs exert multifaceted biological effects, encompassing reductions in blood glucose concentrations, improvements in insulin sensitivity, modulation of metabolic pathways, and reshaping of intestinal microbiota composition. These findings provide a robust scientific basis for the potential application of GELNs in preventive strategies against prediabetes. Nevertheless, the precise molecular mechanisms underlying these beneficial outcomes require further comprehensive investigation. Moreover, the cross‐species regulatory properties of GELNs offer a conceptual framework for the development of novel plant‐derived nanomedicines.

However, the sample size in the present study was relatively small (*n* = 6 per group), which may have limited the statistical power of the microbiota analyses and introduced constraints in the interpretation of the results. Although statistically significant differences were observed for key outcome measures, the limited sample size could have influenced the robustness of microbial community analyses, including PCoA clustering patterns, which may be susceptible to random variation. Therefore, future studies should employ larger cohorts to enhance statistical power and ensure the reliability and reproducibility of the findings. In addition, further mechanistic investigations are needed to elucidate the specific modes of action of GELNs in prediabetes management, thereby providing a stronger scientific basis for potential clinical applications.

In interpreting the present results, we also acknowledge the possible influence of confounding factors, such as batch effects, cage effects, or sequencing biases, which may have affected the microbial community analysis. Although randomization and stringent experimental protocols were implemented to minimize these sources of variability, residual uncertainties may persist in the interpretation of the findings. Subsequent studies should adopt more sophisticated experimental designs and analytical approaches to better quantify and control for such confounding influences.

## Author Contributions

ZLL and SYC conceived the project, designed the study, and drafted the manuscript. SYC, ZZ, and AQ conducted the experiments and carried out the analysis and interpretation of the data. YLY and XDM provided technical or material support.

## Funding

This work was supported by the National Natural Science Foundation of China (10.13039/501100001809) (Grant No. 82160605) and the Xinjiang Uyghur Autonomous Region’s 14th Five‐Year Plan for Characteristic Disciplines in Public Health and Preventive Medicine in Higher Education.

## Disclosure

All authors read and approved the final manuscript.

## Ethics Statement

This study was approved by the Experimental Animal Ethics Committee of Xinjiang Medical University (Approval No. IACUC‐20220720‐25), following the ethical principles of animal experiments to ensure animal welfare. All experimental operations were carried out in accordance with the approved protocol, strictly following humanitarian principles.

## Conflicts of Interest

The authors declare no conflicts of interest.

## Supporting information


**Supporting Information** Additional supporting information can be found online in the Supporting Information section. The supplementary materials accompanying this study provide additional supporting data and visualizations for key analyses. Figure S1: Pathway mapping diagrams for the three metabolic pathways investigated. Figure S2: Functional annotation analysis based on the Clusters of Orthologous Groups (COG) database revealing significant differences in several predicted functional categories between the GELN intervention group (Group W) and the model group (Group T), including exonuclease VII small subunit, leucyl aminopeptidase (aminopeptidase T), and the predicted nucleotide‐utilizing enzyme MoeA. Table S1: Detailed study design.

## Data Availability

Data sharing is not applicable to this article, as no datasets were generated or analyzed during the current study.

## References

[1] Tabák A. G. , Herder C. , Rathmann W. , Brunner E. J. , and Kivimäki M. , Prediabetes: A High-Risk State for Diabetes Development, Lancet. (2012) 379, no. 9833, 2279–2290, 10.1016/S0140-6736(12)60283-9, 22683128. 22683128 PMC3891203

[2] Sun H. , Saeedi P. , Karuranga S. , Pinkepank M. , Ogurtsova K. , Duncan B. B. , Stein C. , Basit A. , Chan J. C. N. , Mbanya J. C. , Pavkov M. E. , Ramachandaran A. , Wild S. H. , James S. , Herman W. H. , Zhang P. , Bommer C. , Kuo S. , Boyko E. J. , and Magliano D. J. , IDF Diabetes Atlas: Global, Regional and Country-Level Diabetes Prevalence Estimates for 2021 and Projections for 2045, Diabetes Research and Clinical Practice. (2022) 183, 109119, 10.1016/j.diabres.2021.109119, 34879977. 34879977 PMC11057359

[3] Echouffo-Tcheugui J. B. and Selvin E. , Prediabetes and What It Means: The Epidemiological Evidence, Annual Review of Public Health. (2021) 42, no. 1, 59–77, 10.1146/annurev-publhealth-090419-102644, 33355476. PMC8026645 33355476

[4] Ansari P. , Akther S. , Hannan J. M. A. , Seidel V. , Nujat N. J. , and Abdel-Wahab Y. H. A. , Pharmacologically Active Phytomolecules Isolated From Traditional Antidiabetic Plants and Their Therapeutic Role for the Management of Diabetes Mellitus, Molecules. (2022) 27, no. 13, 10.3390/molecules27134278, 35807526. PMC9268530 35807526

[5] Majhi S. , Singh L. , Verma M. , Chauhan I. , kumari R. , and Sharma M. , In-Vivo Evaluation and Formulation Development of Polyherbal Extract in Streptozotocin-Induced Diabetic Rat, Phytomedicine Plus. (2022) 2, no. 4, 100337, 10.1016/j.phyplu.2022.100337.

[6] Li H. , Weng Q. , Gong S. , Zhang W. , Wang J. , Huang Y. , Li Y. , Guo J. , and Lan T. , Kaempferol Prevents Acetaminophen-Induced Liver Injury by Suppressing Hepatocyte Ferroptosis via Nrf2 Pathway Activation, Food & Function. (2023) 14, no. 4, 1884–1896, 10.1039/D2FO02716J, 36723004. 36723004

[7] Zhang W. J. , Li Y. Y. , Xiang Z. H. , Deng J. , Li W. , Lin Q. L. , Fang Y. , Liu F. , Bai J. , Zhang L. , and Li J. , Emerging Evidence on the Effects of Plant-Derived MicroRNAs in Colorectal Cancer: A Review, Food & Function. (2023) 14, no. 2, 691–702, 10.1039/D2FO03477H, 36625207. 36625207

[8] Martino E. , D’onofrio N. , Balestrieri A. , Colloca A. , Anastasio C. , Sardu C. , Marfella R. , Campanile G. , and Balestrieri M. L. , Dietary Epigenetic Modulators: Unravelling the Still-Controversial Benefits of miRNAs in Nutrition and Disease, Nutrients. (2024) 16, no. 1, 10.3390/nu16010160, 38201989. PMC10780859 38201989

[9] Sivri D. and Gezmen-Karadağ M. , Effects of Phytochemicals on Type 2 Diabetes via MicroRNAs, Current Nutrition Reports. (2024) 13, no. 3, 444–454, 10.1007/s13668-024-00549-5, 38805166. 38805166 PMC11327184

[10] Liu C. , Xu M. , Yan L. , Wang Y. , Zhou Z. , Wang S. , Sun Y. , Zhang J. , and Dong L. , Honeysuckle-Derived MicroRNA2911 Inhibits Tumor Growth by Targeting TGF-*β*1, Chinese Medicine. (2021) 16, no. 1, 10.1186/s13020-021-00453-y, 34187513. PMC8244210 34187513

[11] Fu B. , Xie J. , Kaneko G. , Wang G. , Yang H. , Tian J. , Xia Y. , Li Z. , Gong W. , Zhang K. , and Yu E. , MicroRNA-Dependent Regulation of Targeted mRNAs for Improved Muscle Texture in Crisp Grass Carp Fed With Broad Bean, Food Research International. (2022) 155, 111071, 10.1016/j.foodres.2022.111071, 35400449. 35400449

[12] Shi L. , Guo C. , Fang M. , Yang Y. , Yin F. , and Shen Y. , Cross-Kingdom Regulation of Plant MicroRNAs: Potential Application in Crop Improvement and Human Disease Therapeutics, Frontiers in Plant Science. (2024) 15, 1512047, 10.3389/fpls.2024.1512047, 39741676. 39741676 PMC11685121

[13] Sabana A. A. , Rajesh M. K. , and Antony G. , Dynamic Changes in the Expression Pattern of miRNAs and Associated Target Genes During Coconut Somatic Embryogenesis, Planta. (2020) 251, no. 4, 10.1007/s00425-020-03368-4, 32166498. 32166498

[14] Zhang J. , Tian S. , Guo L. , Zhao H. , Mao Z. , and Miao M. , Chinese Herbal Medicine-Derived Extracellular Vesicles as Novel Biotherapeutic Tools: Present and Future, Journal of Translational Medicine. (2024) 22, no. 1, 10.1186/s12967-024-05892-3, 39587576. PMC11587639 39587576

[15] Yan L. , Cao Y. , Hou L. , Luo T. , Li M. , Gao S. , Wang L. , Sheng K. , and Zheng L. , Ginger Exosome-Like Nanoparticle-Derived miRNA Therapeutics: A Strategic Inhibitor of Intestinal Inflammation, Journal of Advanced Research. (2025) 69, 1–15, 10.1016/j.jare.2024.04.001, 38588850. 38588850 PMC11954804

[16] Zu M. , Xie D. , Canup B. S. B. , Chen N. , Wang Y. , Sun R. , Zhang Z. , Fu Y. , Dai F. , and Xiao B. , ‘Green’ Nanotherapeutics From Tea Leaves for Orally Targeted Prevention and Alleviation of Colon Diseases, Biomaterials. (2021) 279, 121178, 10.1016/j.biomaterials.2021.121178, 34656857. 34656857

[17] Yang M. , Liu X. , Luo Q. , Xu L. , and Chen F. , An Efficient Method to Isolate Lemon Derived Extracellular Vesicles for Gastric Cancer Therapy, Journal of Nanobiotechnology. (2020) 18, no. 1, 10.1186/s12951-020-00656-9, 32690102. PMC7370524 32690102

[18] Kaur P. , Kotru S. , Singh S. , Behera B. S. , and Munshi A. , Role of miRNAs in the Pathogenesis of T2DM, Insulin Secretion, Insulin Resistance, and *β* Cell Dysfunction: The Story So Far, Journal of Physiology and Biochemistry. (2020) 76, no. 4, 485–502, 10.1007/s13105-020-00760-2, 32749641. 32749641

[19] Yang Fei H. Z. L. S. Q. L. J. H. , Clinical Efficacy of *Ginkgo biloba* Extract on Diabetic Kidney Disease and Its Influence on Urinary Exosome MicroRNA-342-3p, Chinese Journal of Critical Care Medicine (Electronic Edition). (2024) 17, no. 3, 219–224, 10.3877/cma.j.issn.1674-6880.2024.03.007.

[20] Wu B. , Pan W. , Luo S. , Luo X. , Zhao Y. , Xiu Q. , Zhong M. , Wang Z. , Liao T. , Li N. , Liu C. , Nie C. , Yi G. , Lin S. , Zou M. , Li B. , and Zheng L. , Turmeric-Derived Nanoparticles Functionalized Aerogel Regulates Multicellular Networks to Promote Diabetic Wound Healing, Advanced Science. (2024) 11, no. 18, e2307630, 10.1002/advs.202307630, 38441389. 38441389 PMC11095230

[21] Li Y. , Zheng Z. , Kong X. , Wang Y. , Liu Z. , Wang W. , Hu H. , Xu F. , and Shi Y. , Bacteria Extracellular Vesicles Derived fromLactobacillus reuteridelivering Intrinsic miR-21a-5p to Accelerate Diabetic Wound Healing, Nano Research. (2025) 18, no. 11, 94908083, 10.26599/NR.2025.94908083.

[22] Jin E. , Yang Y. , Cong S. , Chen D. , Chen R. , Zhang J. , Hu Y. , and Chen W. , Lemon-Derived Nanoparticle-Functionalized Hydrogels Regulate Macrophage Reprogramming to Promote Diabetic Wound Healing, Journal of Nanobiotechnology. (2025) 23, no. 1, 10.1186/s12951-025-03138-y, 39891270. PMC11783766 39891270

[23] Suresh A. , Ravilla J. , Narayanan J. , and Sundaram G. M. , Mango Ginger-Derived Exosome-Like Nanovesicles Promotes Diabetic Wound Healing via Inducing the Promigratory Protein, Follistatin-Like 1, International Journal of Biological Macromolecules. (2025) 322, Part 4, 146991, 10.1016/j.ijbiomac.2025.146991, 40846023. 40846023

[24] Ju S. , Mu J. , Dokland T. , Zhuang X. , Wang Q. , Jiang H. , Xiang X. , Deng Z. B. , Wang B. , Zhang L. , Roth M. , Welti R. , Mobley J. , Jun Y. , Miller D. , and Zhang H. G. , Grape Exosome-Like Nanoparticles Induce Intestinal Stem Cells and Protect Mice From DSS-Induced Colitis, Molecular Therapy. (2013) 21, no. 7, 1345–1357, 10.1038/mt.2013.64, 23752315. 23752315 PMC3702113

[25] Aragão M. Â. , Pires L. , Santos-Buelga C. , Barros L. , and Calhelha R. C. , Revitalising Riboflavin: Unveiling Its Timeless Significance in Human Physiology and Health, Foods. (2024) 13, no. 14, 10.3390/foods13142255, 39063339. PMC11276209 39063339

[26] Di Lorenzo C. , Colombo F. , Biella S. , Stockley C. , and Restani P. , Polyphenols and Human Health: The Role of Bioavailability, Nutrients. (2021) 13, no. 1, 10.3390/nu13010273, 33477894. PMC7833401 33477894

[27] Chen C. , Wang J. , Pan D. , Wang X. , Xu Y. , Yan J. , Wang L. , Yang X. , Yang M. , and Liu G. P. , Applications of Multi-Omics Analysis in Human Diseases, MedComm. (2023) 4, no. 4, e315, 10.1002/mco2.315. 37533767 PMC10390758

[28] Agbu P. and Carthew R. W. , MicroRNA-Mediated Regulation of Glucose and Lipid Metabolism, Nature Reviews Molecular Cell Biology. (2021) 22, no. 6, 425–438, 10.1038/s41580-021-00354-w, 33772227. 33772227 PMC8853826

[29] Dexheimer P. J. and Cochella L. , MicroRNAs: From Mechanism to Organism, Frontiers in Cell and Developmental Biology. (2020) 8, 10.3389/fcell.2020.00409, 32582699. PMC7283388 32582699

[30] Fan Y. and Pedersen O. , Gut Microbiota in Human Metabolic Health and Disease, Nature Reviews Microbiology. (2021) 19, no. 1, 55–71, 10.1038/s41579-020-0433-9, 32887946. 32887946

[31] Ahlberg E. , Al-Kaabawi A. , Thune R. , Simpson M. R. , Pedersen S. A. , Cione E. , Jenmalm M. C. , and Tingö L. , Breast Milk MicroRNAs: Potential Players in Oral Tolerance Development, Frontiers in Immunology. (2023) 14, 1154211, 10.3389/fimmu.2023.1154211, 36999032. 36999032 PMC10045994

[32] Jiang S. , Research on the Correlation Between Antioxidant Dietary Patterns Extracted by Reduced-Rank Regression Method and Type 2 Diabetes, Xinjiang Medical University, 2023.

[33] Chen Xuan , Chen X. , and Wang Shaoyun , The Intervention Effect of Total Flavonoids Extract from Vine Tea on Prediabetic Mice, Food Science. (2019) 40, no. 5.

[34] Kim J. , Li S. , Zhang S. , and Wang J. , Plant-Derived Exosome-Like Nanoparticles and Their Therapeutic Activities, Asian Journal of Pharmaceutical Sciences. (2022) 17, no. 1, 53–69, 10.1016/j.ajps.2021.05.006, 35261644. 35261644 PMC8888139

[35] Shaheen N. , Shaheen A. , Diab R. A. , and Desouki M. T. , MicroRNAs (miRNAs) Role in Hypertension: Pathogenesis and Promising Therapeutics, Annals of Medicine and Surgery. (2024) 86, no. 1, 319–328, 10.1097/MS9.0000000000001498, 38222760. 38222760 PMC10783350

[36] Mu N. , Li J. , Zeng L. , You J. , Li R. , Qin A. , Liu X. , Yan F. , and Zhou Z. , Plant-Derived Exosome-Like Nanovesicles: Current Progress and Prospects, International Journal of Nanomedicine. (2023) 18, 4987–5009, 10.2147/IJN.S420748, 37693885. 37693885 PMC10492547

[37] He C. , Wang K. , Xia J. , Qian D. , Guo J. , Zhong L. , Tang D. , Chen X. , Peng W. , Chen Y. , and Tang Y. , Natural Exosomes-Like Nanoparticles in Mung Bean Sprouts Possesses Anti-Diabetic Effects via Activation of PI3K/Akt/GLUT4/GSK-3*β* Signaling Pathway, Journal of Nanobiotechnology. (2023) 21, no. 1, 10.1186/s12951-023-02120-w, 37759297. PMC10536756 37759297

[38] Wang W. , Zhu N. , Yan T. , Shi Y. N. , Chen J. , Zhang C. J. , Xie X. J. , Liao D. F. , and Qin L. , The Crosstalk: Exosomes and Lipid Metabolism, Cell Communication and Signaling. (2020) 18, no. 1, 10.1186/s12964-020-00581-2, 32746850. PMC7398059 32746850

[39] Wang Z. , Zhang J. , Zhang S. , Yan S. , Wang Z. , Wang C. , and Zhang X. , miR-30e and miR-92a Are Related to Atherosclerosis by Targeting ABCA1, Molecular Medicine Reports. (2019) 19, no. 4, 3298–3304, 10.3892/mmr.2019.9983, 30816508. 30816508

[40] Nikravesh M. , Mahdavinia M. , Neisi N. , Khorsandi L. , and Khodayar M. J. , Citicoline Ameliorates Arsenic-Induced Hepatotoxicity and Diabetes in Mice by Overexpression of VAMP2, PPAR-*γ*, As3MT, and SIRT3, Pesticide Biochemistry and Physiology. (2023) 192, 105391, 10.1016/j.pestbp.2023.105391, 37105618. 37105618

[41] Ma Z. , Du B. , Li J. , Yang Y. , and Zhu F. , An Insight Into Anti-Inflammatory Activities and Inflammation Related Diseases of Anthocyanins: A Review of Both In Vivo and In Vitro Investigations, International Journal of Molecular Sciences. (2021) 22, no. 20, 11076, 10.3390/ijms222011076, 34681733. 34681733 PMC8540239

[42] Tuell D. , Ford G. , Los E. , and Stone W. , The Role of Glutathione and Its Precursors in Type 2 Diabetes, Antioxidants. (2024) 13, no. 2, 10.3390/antiox13020184. PMC10885928 38397782

[43] Xue C. , Li G. , Zheng Q. , Gu X. , Shi Q. , Su Y. , Chu Q. , Yuan X. , Bao Z. , Lu J. , and Li L. , Tryptophan Metabolism in Health and Disease, Cell Metabolism. (2023) 35, no. 8, 1304–1326, 10.1016/j.cmet.2023.06.004. 37352864

[44] Gao J. , Yang T. , Song B. , Ma X. , Ma Y. , Lin X. , and Wang H. , Abnormal Tryptophan Catabolism in Diabetes Mellitus and Its Complications: Opportunities and Challenges, Biomedicine and Pharmacotherapy. (2023) 166, 115395, 10.1016/j.biopha.2023.115395, 37657259. 37657259

[45] Mosegaard S. , Dipace G. , Bross P. , Carlsen J. , Gregersen N. , and Olsen R. K. J. , Riboflavin Deficiency-Implications for General Human Health and Inborn Errors of Metabolism, International Journal of Molecular Sciences. (2020) 21, no. 11, 10.3390/ijms21113847, 32481712. PMC7312377 32481712

[46] Sokol H. , Pigneur B. , Watterlot L. , Lakhdari O. , Bermúdez-Humarán L. G. , Gratadoux J. J. , Blugeon S. , Bridonneau C. , Furet J. P. , Corthier G. , Grangette C. , Vasquez N. , Pochart P. , Trugnan G. , Thomas G. , Blottière H. M. , Doré J. , Marteau P. , Seksik P. , and Langella P. , *Faecalibacterium prausnitzii* Is an Anti-Inflammatory Commensal Bacterium Identified by Gut Microbiota Analysis of Crohn Disease Patients, Proceedings of the National Academy of Sciences of the United States of America. (2008) 105, no. 43, 16731–16736, 10.1073/pnas.0804812105, 18936492. 18936492 PMC2575488

[47] González Hernández M. A. , Canfora E. E. , Jocken J. W. , and Blaak E. E. , The Short-Chain Fatty Acid Acetate in Body Weight Control and Insulin Sensitivity, Nutrients. (2019) 11, no. 8, 10.3390/nu11081943. PMC6723943 31426593

[48] Amar J. , Chabo C. , Waget A. , Klopp P. , Vachoux C. , Bermúdez-Humarán L. G. , Smirnova N. , Bergé M. , Sulpice T. , Lahtinen S. , Ouwehand A. , Langella P. , Rautonen N. , Sansonetti P. J. , and Burcelin R. , Intestinal Mucosal Adherence and Translocation of Commensal Bacteria at the Early Onset of Type 2 Diabetes: Molecular Mechanisms and Probiotic Treatment, EMBO Molecular Medicine. (2011) 3, no. 9, 559–572, 10.1002/emmm.201100159, 21735552. 21735552 PMC3265717

[49] Hänninen A. , Toivonen R. , Pöysti S. , Belzer C. , Plovier H. , Ouwerkerk J. P. , Emani R. , Cani P. D. , and de Vos W. M. , *Akkermansia muciniphila* Induces Gut Microbiota Remodelling and Controls Islet Autoimmunity in NOD Mice, Gut. (2018) 67, no. 8, 1445–1453, 10.1136/gutjnl-2017-314508, 29269438. 29269438

[50] Zhao L. , Zhang F. , Ding X. , Wu G. , Lam Y. Y. , Wang X. , Fu H. , Xue X. , Lu C. , Ma J. , Yu L. , Xu C. , Ren Z. , Xu Y. , Xu S. , Shen H. , Zhu X. , Shi Y. , Shen Q. , Dong W. , Liu R. , Ling Y. , Zeng Y. , Wang X. , Zhang Q. , Wang J. , Wang L. , Wu Y. , Zeng B. , Wei H. , Zhang M. , Peng Y. , and Zhang C. , Gut Bacteria Selectively Promoted by Dietary Fibers Alleviate Type 2 Diabetes, Science. (2018) 359, no. 6380, 1151–1156, 10.1126/science.aao5774, 29590046. 29590046

[51] Tian T. , Zhang X. , Luo T. , Wang D. , Sun Y. , and Dai J. , Effects of Short-Term Dietary Fiber Intervention on Gut Microbiota in Young Healthy People, Diabetes, Metabolic Syndrome and Obesity. (2021) 14, 3507–3516, 10.2147/DMSO.S313385, 34385825. PMC8353528 34385825

[52] Zhou X. , Liu W. , Gu M. , Zhou H. , and Zhang G. , *Helicobacter pylori* Infection Causes Hepatic Insulin Resistance by the c-Jun/miR-203/SOCS3 Signaling Pathway, Journal of Gastroenterology. (2015) 50, no. 10, 1027–1040, 10.1007/s00535-015-1051-6, 25689935. 25689935

[53] Talebi G. , Saffarian P. , Hakemi-Vala M. , Sadeghi A. , and Yadegar A. , The Effect of *Helicobacter pylori*-Derived Extracellular Vesicles on Glucose Metabolism and Induction of Insulin Resistance in HepG2 Cells, Archives of Physiology and Biochemistry. (2025) 131, no. 2, 316–327, 10.1080/13813455.2024.2418494, 39431628. 39431628

[54] Niu H. , Zhou M. , Zogona D. , Xing Z. , Wu T. , Chen R. , Cui D. , Liang F. , and Xu X. , *Akkermansia muciniphila*: A Potential Candidate for Ameliorating Metabolic Diseases, Frontiers in Immunology. (2024) 15, 10.3389/fimmu.2024.1370658, 38571945. PMC10987721 38571945

[55] Zeng Z. , Chen M. , Liu Y. , Zhou Y. , Liu H. , Wang S. , and Ji Y. , Role ofAkkermansia muciniphilain Insulin Resistance, Journal of Gastroenterology and Hepatology. (2025) 40, no. 1, 19–32, 10.1111/jgh.16747. 39396929

[56] Teng Y. , Ren Y. , Sayed M. , Hu X. , Lei C. , Kumar A. , Hutchins E. , Mu J. , Deng Z. , Luo C. , Sundaram K. , Sriwastva M. K. , Zhang L. , Hsieh M. , Reiman R. , Haribabu B. , Yan J. , Jala V. R. , Miller D. M. , van Keuren-Jensen K. , Merchant M. L. , McClain C. J. , Park J. W. , Egilmez N. K. , and Zhang H. G. , Plant-Derived Exosomal MicroRNAs Shape the Gut Microbiota, Cell Host & Microbe. (2018) 24, no. 5, 637–652.e8, 10.1016/j.chom.2018.10.001, 30449315. 30449315 PMC6746408

[57] Khalili L. , Park G. , Nagpal R. , and Salazar G. , The Role of *Akkermansia muciniphila* on Improving Gut and Metabolic Health Modulation: A Meta-Analysis of Preclinical Mouse Model Studies, Microorganisms. (2024) 12, no. 8, 10.3390/microorganisms12081627, 39203469. PMC11356609 39203469

[58] Mo C. , Lou X. , Xue J. , Shi Z. , Zhao Y. , Wang F. , and Chen G. , The Influence of *Akkermansia muciniphila* on Intestinal Barrier Function, Gut Pathogens. (2024) 16, no. 1, 10.1186/s13099-024-00635-7, 39097746. PMC11297771 39097746

[59] Xuan W. , Ou Y. , Chen W. , Huang L. , Wen C. , Huang G. , Tang W. , Zeng D. , Huang S. , Xiao L. , and Li Z. , *Faecalibacterium prausnitzii* Improves Lipid Metabolism Disorder and Insulin Resistance in Type 2 Diabetic Mice, British Journal of Biomedical Science. (2023) 80, 10794, 10.3389/bjbs.2023.10794, 37025162. 37025162 PMC10070466

[60] Zhong H. , Wang L. , Jia F. , Yan Y. , Xiong F. , Li Y. , Hidayat K. , and Guan R. , Effects of *Lactobacillus plantarum* Supplementation on Glucose and Lipid Metabolism in Type 2 Diabetes Mellitus and Prediabetes: A Systematic Review and Meta-Analysis of Randomized Controlled Trials, Clinical Nutrition ESPEN. (2024) 61, 377–384, 10.1016/j.clnesp.2024.04.009, 38777458. 38777458

[61] Boronovskiy S. E. , Kopylova V. S. , and Nartsissov Y. R. , Metabolism and Receptor Mechanisms of Niacin Action, Cell and Tissue Biology. (2024) 18, no. 2, 128–147, 10.1134/S1990519X23700025.

