# Supplementary material for: Grape Exosome–Like Nanovesicles Reverse the Prediabetic State in Mice
Source: J Diabetes Res. 2026 May 31;2026:6667696. doi: 10.1155/jdr/6667696 (PMC13239045; doi:10.1155/jdr/6667696)
Supplement: Supplementary file 1 — Supporting Information Additional supporting information can be found online in the Supporting Information section. The supplementary materials accompanying this study provide additional supporting data and visualizations for key analyses. Figure S1: Pathway mapping diagrams for the three metabolic pathways investigated. Figure S2: Functional annotation analysis based on the Clusters of Orthologous Groups (COG) database revealing significant differences in several predicted functional categories between the GELN intervention group (Group W) and the model group (Group T), including exonuclease VII small subunit, leucyl aminopeptidase (aminopeptidase T), and the predicted nucleotide‐utilizing enzyme MoeA. Table S1: Detailed study design. [file JDR-2026-6667696-s001.zip › 6667696.f1/Supplementary Material Table 1.docx]

Supplementary Material Table 1 Detailed Experimental Design

| Group name | Processing method | ime point | Specific operation | Intervention dose |
| --- | --- | --- | --- | --- |
| Control group | Normal diet | Week 1 - Week 12 | Normal diet, without any intervention | — |
| Model group | High-sugar and high-fat diet + STZ injection | Week 1 - Week 3 | Feeding with high-fat diet (60% high-fat diet) | — |
|  |  | The end of the third week | Intraperitoneal injection of STZ (60 mg/kg, under a dark ice bath, prepared immediately as needed, and used up within 30 minutes) | — |
|  |  | Day 7, Day 21 | Conduct an OGTT test (fasting blood glucose < 7.8 mmol/L, blood glucose 2 hours after meal 7.8 - 11.1 mmol/L) | — |
|  |  | Week 4 - Week 12 | Continuous high-fat feed feeding without any intervention | — |
| The GELNs intervention group | High-sugar and high-fat diet + STZ injection | Week 1 - Week 3 | Feeding with high-fat diet (60% high-fat diet) | — |
|  |  | The end of the third week | Intraperitoneal injection of STZ (60 mg/kg, under a dark ice bath, prepared immediately as needed, and used up within 30 minutes) | — |
|  |  | Day 7, Day 21 | Conduct an OGTT test (fasting blood glucose < 7.8 mmol/L, blood glucose 2 hours after meal 7.8 - 11.1 mmol/L) | — |
|  |  | Week 4 - Week 12 | Continuous high-fat diet feeding, intragastric administration of GELNs daily | 0.5 mg/ml |
| Nutrient intervention group | High-sugar and high-fat diet + STZ injection | Week 1 - Week 3 | Feeding with high-fat diet (60% high-fat diet) | — |
|  |  | The end of the third week | Intraperitoneal injection of STZ (60 mg/kg, under a dark ice bath, prepared immediately as needed, and used up within 30 minutes) | — |
|  |  | Day 7, Day 21 | Conduct an OGTT test (fasting blood glucose < 7.8 mmol/L, blood glucose 2 hours after meal 7.8 - 11.1 mmol/L) | — |
|  |  | Week 4 - Week 12 | Continuous high-fat feed feeding, daily intragastric administration of nutrients (vitamin C, vitamin E, selenium) | Vitamin C: 15.17 mg/kg;  Vitamin E: 2.12 mg/kg; Selenium: 9.1 μg/kg |
