# Supplementary figures and images for: Grape Exosome–Like Nanovesicles Reverse the Prediabetic State in Mice
Source: J Diabetes Res. 2026 May 31;2026:6667696. doi: 10.1155/jdr/6667696 (PMC13239045; doi:10.1155/jdr/6667696)

## Slide 1
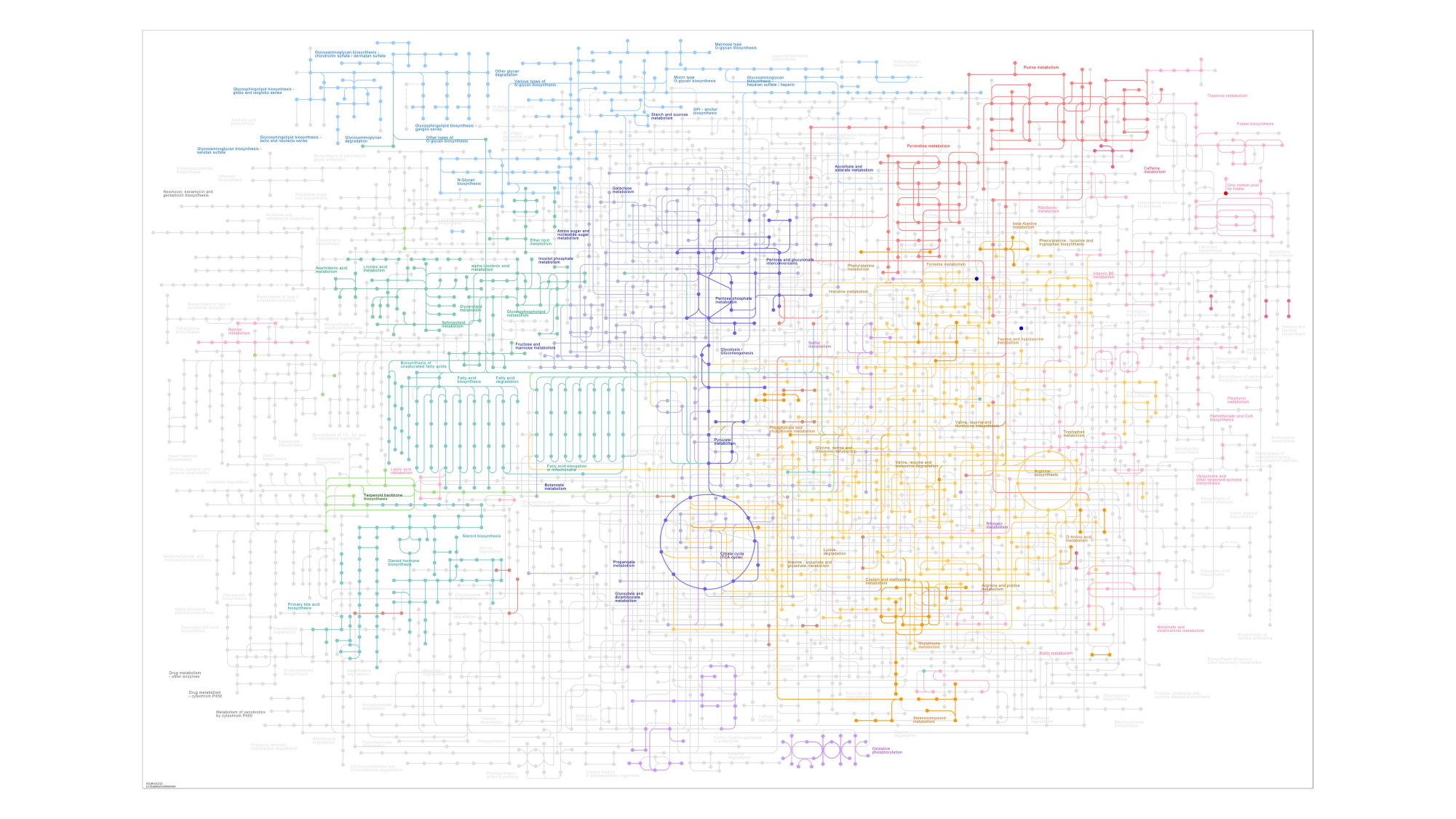

Supplement: Supplementary file 1 — Supporting Information Additional supporting information can be found online in the Supporting Information section. The supplementary materials accompanying this study provide additional supporting data and visualizations for key analyses. Figure S1: Pathway mapping diagrams for the three metabolic pathways investigated. Figure S2: Functional annotation analysis based on the Clusters of Orthologous Groups (COG) database revealing significant differences in several predicted functional categories between the GELN intervention group (Group W) and the model group (Group T), including exonuclease VII small subunit, leucyl aminopeptidase (aminopeptidase T), and the predicted nucleotide‐utilizing enzyme MoeA. Table S1: Detailed study design. [file JDR-2026-6667696-s001.zip › 6667696.f1/Supplementary Material Fig 1A.pptx]

## Slide 1
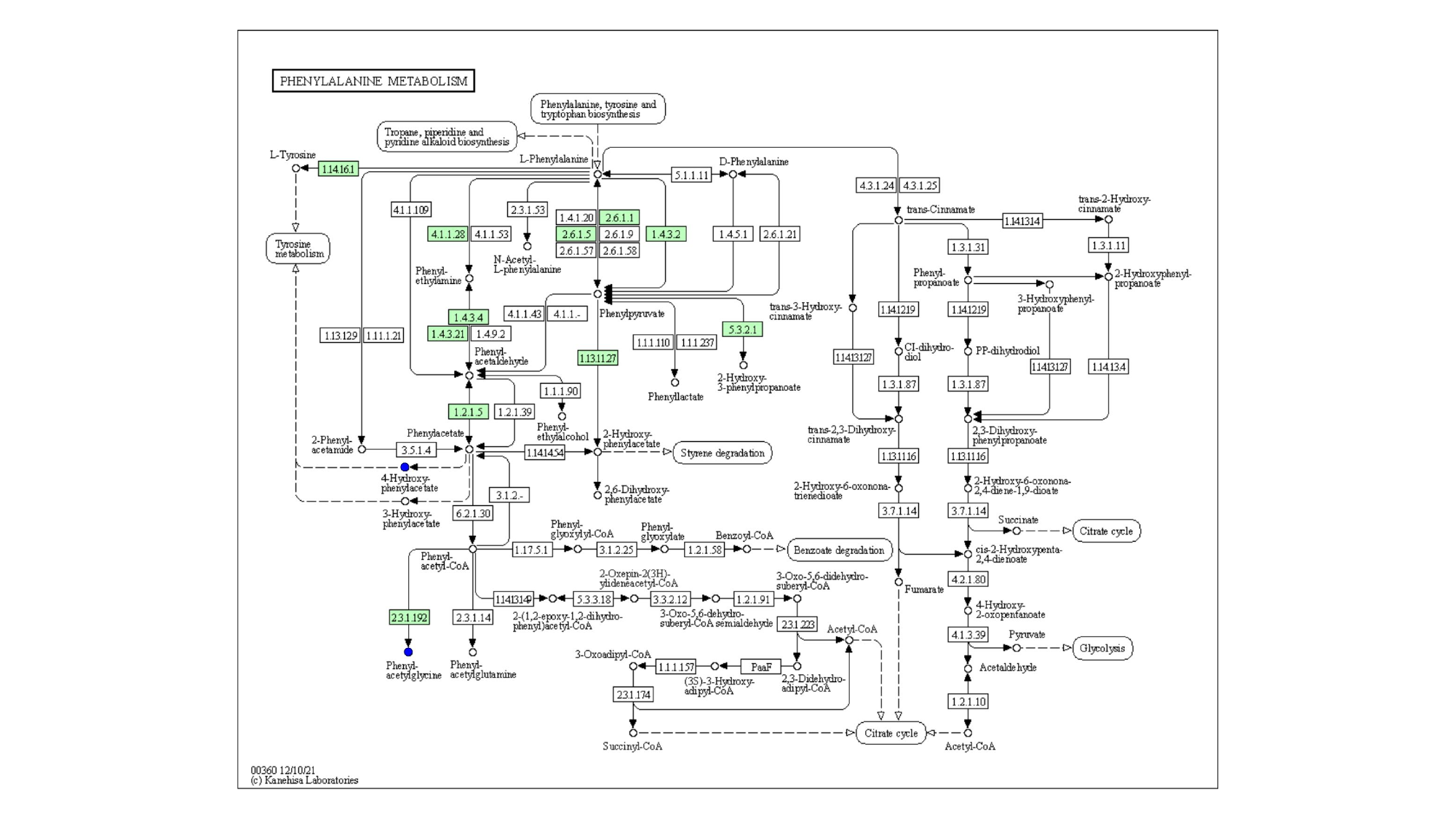

Supplement: Supplementary file 1 — Supporting Information Additional supporting information can be found online in the Supporting Information section. The supplementary materials accompanying this study provide additional supporting data and visualizations for key analyses. Figure S1: Pathway mapping diagrams for the three metabolic pathways investigated. Figure S2: Functional annotation analysis based on the Clusters of Orthologous Groups (COG) database revealing significant differences in several predicted functional categories between the GELN intervention group (Group W) and the model group (Group T), including exonuclease VII small subunit, leucyl aminopeptidase (aminopeptidase T), and the predicted nucleotide‐utilizing enzyme MoeA. Table S1: Detailed study design. [file JDR-2026-6667696-s001.zip › 6667696.f1/Supplementary Material Fig 1B.pptx]

## Slide 1
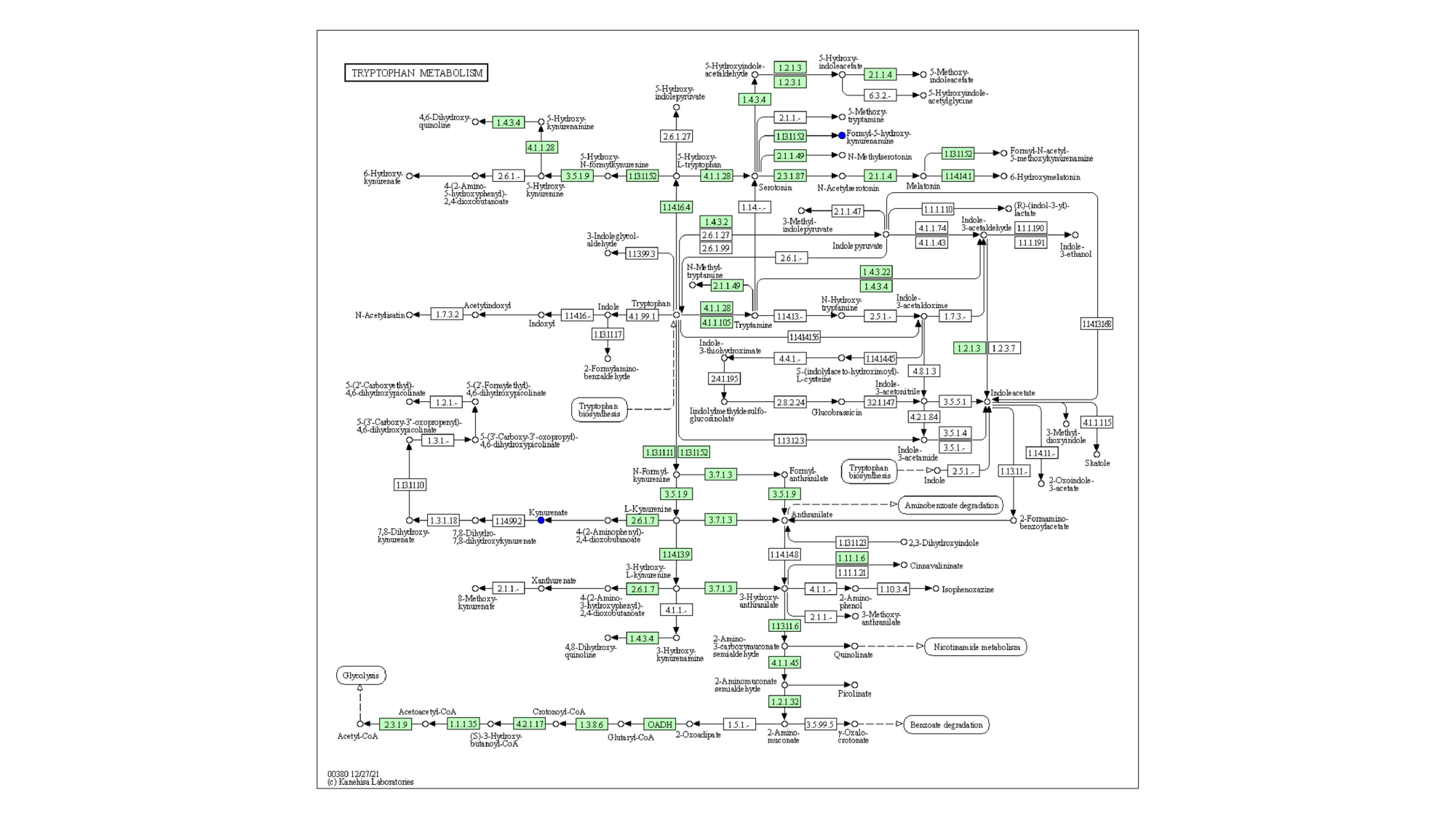

Supplement: Supplementary file 1 — Supporting Information Additional supporting information can be found online in the Supporting Information section. The supplementary materials accompanying this study provide additional supporting data and visualizations for key analyses. Figure S1: Pathway mapping diagrams for the three metabolic pathways investigated. Figure S2: Functional annotation analysis based on the Clusters of Orthologous Groups (COG) database revealing significant differences in several predicted functional categories between the GELN intervention group (Group W) and the model group (Group T), including exonuclease VII small subunit, leucyl aminopeptidase (aminopeptidase T), and the predicted nucleotide‐utilizing enzyme MoeA. Table S1: Detailed study design. [file JDR-2026-6667696-s001.zip › 6667696.f1/Supplementary Material Fig 1C.pptx]

## Slide 1
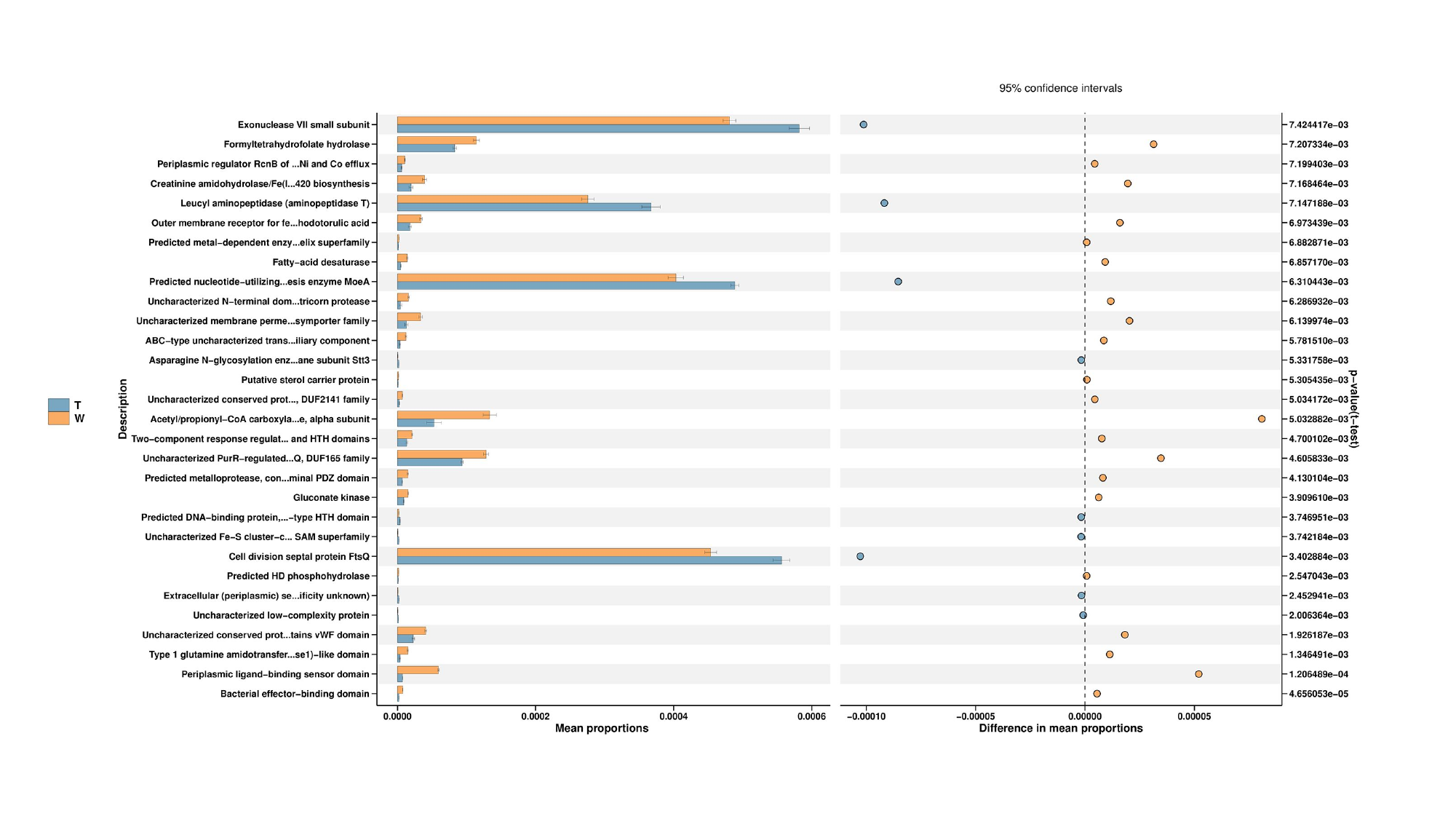

Supplement: Supplementary file 1 — Supporting Information Additional supporting information can be found online in the Supporting Information section. The supplementary materials accompanying this study provide additional supporting data and visualizations for key analyses. Figure S1: Pathway mapping diagrams for the three metabolic pathways investigated. Figure S2: Functional annotation analysis based on the Clusters of Orthologous Groups (COG) database revealing significant differences in several predicted functional categories between the GELN intervention group (Group W) and the model group (Group T), including exonuclease VII small subunit, leucyl aminopeptidase (aminopeptidase T), and the predicted nucleotide‐utilizing enzyme MoeA. Table S1: Detailed study design. [file JDR-2026-6667696-s001.zip › 6667696.f1/Supplementary Material Fig 2.pptx]
